# HuR-mediated nucleocytoplasmic translocation of HOTAIR relieves its inhibition of osteogenic differentiation and promotes bone formation

**DOI:** 10.1038/s41413-023-00289-2

**Published:** 2023-10-23

**Authors:** Yuheng Li, Weijia Sun, Jianwei Li, Ruikai Du, Wenjuan Xing, Xinxin Yuan, Guohui Zhong, Dingsheng Zhao, Zizhong Liu, Xiaoyan Jin, Junjie Pan, Youyou Li, Qi Li, Guanghan Kan, Xuan Han, Shukuan Ling, Xiqing Sun, Yingxian Li

**Affiliations:** 1 The Key Laboratory of Aerospace Medicine, Ministry of Education, The Fourth Military Medical University, Xi’an, Shaanxi China; 2 State Key Laboratory of Space Medicine Fundamentals and Application, China Astronaut Research and Training Center, Beijing, China; 3 The Center of Space Bio-Medicine, Beijing Institute of Technology, Beijing, China; 4 Medical College of Soochow University, Suzhou, Jiangsu China; 5 Oujiang Laboratory (Zhejiang Lab for Regenerative Medicine, Vision and Brain Health), Wenzhou, Zhejiang China

**Keywords:** Bone, Bone quality and biomechanics

## Abstract

Bone marrow mesenchymal stem cell (BMSC) osteogenic differentiation and osteoblast function play critical roles in bone formation, which is a highly regulated process. Long noncoding RNAs (lncRNAs) perform diverse functions in a variety of biological processes, including BMSC osteogenic differentiation. Although several studies have reported that HOX transcript antisense RNA (HOTAIR) is involved in BMSC osteogenic differentiation, its effect on bone formation in vivo remains unclear. Here, by constructing transgenic mice with BMSC (Prx1-HOTAIR)- and osteoblast (Bglap-HOTAIR)-specific overexpression of HOTAIR, we found that Prx1-HOTAIR and Bglap-HOTAIR transgenic mice show different bone phenotypes in vivo. Specifically, Prx1-HOTAIR mice showed delayed bone formation, while Bglap-HOTAIR mice showed increased bone formation. HOTAIR inhibits BMSC osteogenic differentiation but promotes osteoblast function in vitro. Furthermore, we identified that HOTAIR is mainly located in the nucleus of BMSCs and in the cytoplasm of osteoblasts. HOTAIR displays a nucleocytoplasmic translocation pattern during BMSC osteogenic differentiation. We first identified that the RNA-binding protein human antigen R (HuR) is responsible for HOTAIR nucleocytoplasmic translocation. HOTAIR is essential for osteoblast function, and cytoplasmic HOTAIR binds to miR-214 and acts as a ceRNA to increase Atf4 protein levels and osteoblast function. Bglap-HOTAIR mice, but not Prx1-HOTAIR mice, showed alleviation of bone loss induced by unloading. This study reveals the importance of temporal and spatial regulation of HOTAIR in BMSC osteogenic differentiation and bone formation, which provides new insights into precise regulation as a target for bone loss.

## Introduction

BMSCs are the progenitor cells of multiple mesenchymal cell lineages, including osteocytes, chondrocytes, and adipocytes, and have been widely utilized as an effective treatment for abnormal bone metabolic diseases.^1^ BMSCs have the latent capacity/potential to differentiate into mature osteoblasts, requiring a multitude of complex steps.^2^ Several transcription factors, such as Osterix (Sp7) and Runt-related transcription factor 2 (Runx2), play important roles in initiating the osteogenic differentiation of BMSCs.^3^ ATF4 is the key regulator of osteoblast function and bone formation by promoting osteoblast-specific gene expression.^4^ Transcriptional regulation of the osteogenic differentiation of BMSCs is strictly orchestrated by several key molecular signals, where genes essential for bone formation are expressed sequentially. Although several important cytokines, transcription factors, and noncoding RNAs have been confirmed to be involved in the fine regulation of BMSC osteogenic differentiation, the internal mechanisms remain unclear.

LncRNAs perform diverse functions in a variety of biological processes, including cell proliferation, apoptosis, and differentiation,^5^ and regulate gene expression at both the transcriptional and post-transcriptional levels.^6,7^ The expression of most lncRNAs is likely related to their specific environmental conditions, which show a tissue or cell type-specific expression pattern.^8^ Recent evidence has shown that the function of lncRNAs depends on their localization.^9,10^ Some lncRNAs are recognized as important modulators of nuclear functions and exhibit distinct nuclear localization patterns.^11,12^ Others must be exported to the cytoplasm to carry out their regulatory roles. A series of lncRNA binding proteins have been identified to be responsible for its transportation in different contexts.^13,14^ The complexity of the spatial-temporal expression of lncRNAs accounts for their diverse roles under different physiological and pathological conditions.

HOTAIR, an intergenic lncRNA transcribed from the antisense of HoxC11 and HoxC12 loci,^15^ has important pathophysiological roles in the occurrence of cancer metastasis, liver fibrosis, and neurodegenerative disease.^16–19^ Mechanistically, HOTAIR has been reported to be a protein scaffold that can recruit the polycomb repressive complex 2 (PRC2) complex to alter the H3 modification of chromatin and regulate gene expression^20,21^ or act as a protein chaperone to protect genes from post-translational protein degradation.^22^ HOTAIR can also act as a ceRNA for several miRNAs to regulate cell differentiation, proliferation, and apoptosis.^23–25^ Recently, HOTAIR was reported to underlie region-specific development of adipose tissue.^26^ These findings suggest that HOTAIR plays regulatory roles in both the nucleus and cytoplasm.

Targeted deletion of mouse HOTAIR was reported to lead to derepression of hundreds of genes, resulting in homeotic transformation of the spine and malformation of the metacarpal bone.^27^ This finding suggests that the effect of HOTAIR is closely associated with bone development. HOTAIR has been shown to impact the differentiation of BMSC fate by linking senescence-associated DNA modifications.^28^ Additionally, HOTAIR has been found to inhibit osteogenic differentiation of BMSCs by regulating the Wnt/β-catenin pathway or sponging miR-378g.^29,30^ However, a study revealed that the knockdown of HOTAIR decreases BMP9-induced iMAD cell osteogenic differentiation and ectopic bone formation. Furthermore, a study found that the expression levels of HOTAIR in MSCs of patients with nontraumatic necrosis of the femoral head were approximately threefold higher than those of healthy patients. HOTAIR has been identified to regulate osteogenic differentiation and proliferation through inhibition of miR-17-5p.^31^ Another research team discovered that the level of HOTAIR was decreased in postmenopausal females and that HOTAIR participated in the process of estrogen deficiency-induced osteoblast apoptosis by modulating the miR-138/TIMP1 signaling axis.^32^ While there has been extensive research on the effects of HOTAIR on bone metabolism, little is known about its role in different stages of bone formation.^33^ Moreover, the context-dependent function of HOTAIR in the lineage of bone cells is rarely reported. The precise regulatory role of HOTAIR in the osteogenic differentiation of BMSCs and osteogenesis needs further exploration, particularly given the discrepancies identified in the bone phenotypes of HOTAIR knockout mice.

In this study, we found that HOTAIR levels were decreased in osteoblasts isolated from osteoporotic bone. In contrast, its level was increased or remained unchanged in the BMSCs from these bones. Prx1-specific HOTAIR transgenic mice exhibited developmental retardation. However, osteoblast-specific HOTAIR transgenic mice showed enhanced bone formation. We also demonstrated that HOTAIR inhibits the osteogenic differentiation of BMSCs but promotes osteoblast function in vitro. HOTAIR displays different cytoplasmic and nuclear localization patterns in the process of osteogenic differentiation of BMSCs and osteogenesis. Human antigen R (HuR) directly interacts with HOTAIR and mediates its translocation, which is necessary for the function of HOTAIR in the regulation of osteogenesis. Cytoplasm-localized HOTAIR increased osteoblast function by sponging miR-214 to alleviate *Atf4* translation. This study on the function of HOTAIR in bone provides a paradigm for the capacity of lncRNAs to regulate gene transcription through temporal and spatial changes, highlighting the importance of precise lncRNA-driven therapy for the modulation of bone metabolism.

## Results

### Distinct expression of HOTAIR in BMSCs and osteoblasts

**Fig. 1:**
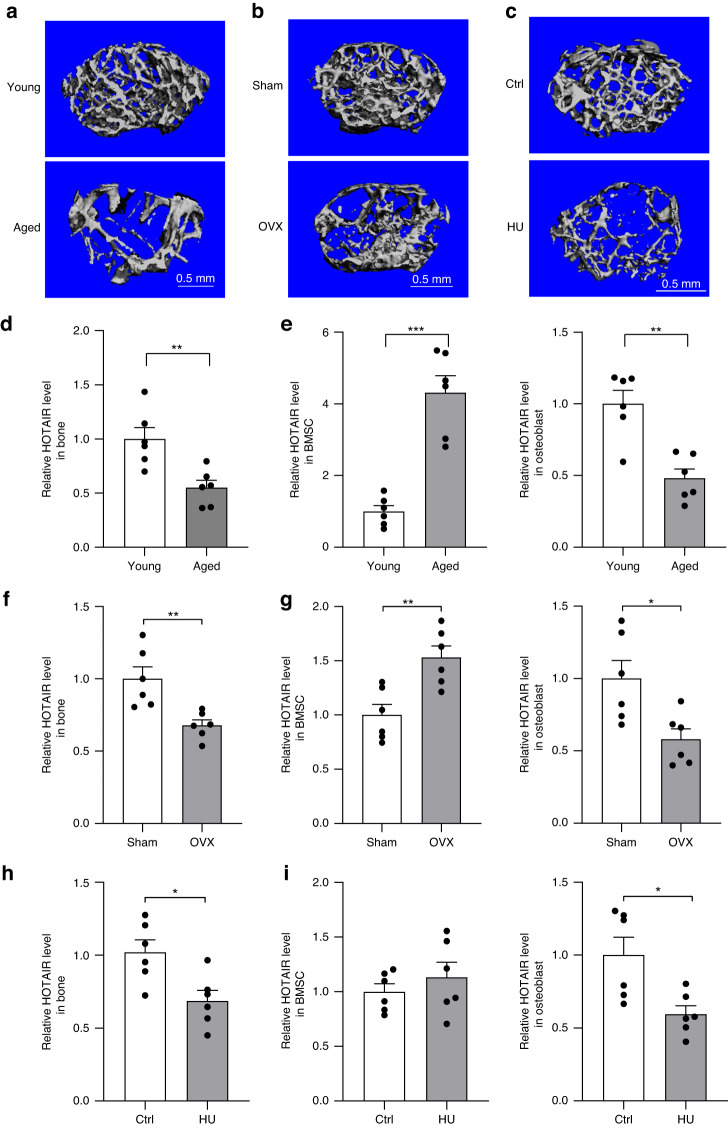
Distinct expression of HOTAIR in BMSCs and osteoblasts. **a** Representative image showing three-dimensional trabecular architecture by micro-CT reconstruction at the distal femurs from young mice (2 months) and aged mice (24 months) (*n* = 6 for each group). Scale bar, 0.5 mm. **b** Representative images showing three-dimensional trabecular architecture by micro-CT reconstruction at the distal femurs from sham- or ovariectomy (OVX)-operated mice for 6 months (*n* = 6 for each group). Scale bar, 0.5 mm. **c** Representative images showing three-dimensional trabecular architecture by micro-CT reconstruction at the distal femurs from mice that were hindlimb unloaded (HU) or control (Ctrl) treated for 28 days (*n* = 6 for each group). Scale bar, 0.5 mm. **d** qRT‒PCR analysis of HOTAIR levels in bone tissue from young and aged mice (*n* = 6 for each group). **e** qRT‒PCR analysis of HOTAIR levels in Sca-1^+^CD29^+^CD45^–^CD11b^–^ bone marrow mesenchymal stem cells (BMSCs) and ALP^+^ osteoblasts separated by cell sorting with fluorescence-activated cell sorting (FACS) from young and aged mice (*n* = 6 for each group). **f** qRT‒PCR analysis of HOTAIR levels in bone tissue from mice that underwent OVX or sham operation (*n* = 6 for each group). **g** qRT‒PCR analysis of HOTAIR levels in bone marrow mesenchymal stem cells (BMSCs) and osteoblasts separated by cell sorting with FACS from OVX and sham mice (*n* = 6 for each group). **h** qRT‒PCR analysis of HOTAIR levels in bone tissue from mice treated with hindlimb unloading (HU) or control (Ctrl) treatment (*n* = 6 for each group). **i** qRT‒PCR analysis of HOTAIR levels in bone marrow mesenchymal stem cells (BMSCs) and osteoblasts separated by cell sorting with FACS from Ctrl and HU mice (*n* = 6 for each group). All data are the mean ± s.e.m. *P < 0.05, **P < 0.01. *** P < 0.001. NS, no significance

To identify the regulation of HOTAIR in bone metabolism and bone tissue cells, we measured the expression of HOTAIR in bone tissue of mice with aging-, ovariectomized (OVX)-, and hindlimb unloading (HU)-induced bone loss. We first detected changes in bone mass, and micro-CT analysis showed that bone mass was decreased in bone tissue from mice with aging-, OVX-, and HU-induced bone loss. The bone structure indices, including bone volume per total volume (BV/TV), bone mineral density (BMD), trabecular number (Tb.N), and trabecular thickness (Tb.Th), of aging mice, OVX mice, and mice with HU were significantly decreased compared with those of young mice, sham mice or Ctrl mice (Fig. 1a–c and Fig. S1a–c). Next, we detected changes in HOTAIR in bone tissue. Sca-1^+^ CD29^+^ CD45^−^ CD11b^−^ BMSCs and ALP^+^ osteoblasts were sorted from three bone loss model mice. The results showed that the level of HOTAIR was significantly decreased in the bone tissue from all three bone loss model mice. The level of HOTAIR was increased in BMSCs and decreased in osteoblasts from aged and OVX mice. and decreased in osteoblasts from mice with HU, although there was no significant difference in BMSCs between the control mice and mice with HU (Fig. 1d-i). The inverse expression patterns of HOTAIR in BMSCs and osteoblasts suggest that HOTAIR plays a different role in BMSCs and osteoblasts.

### HOTAIR overexpression in MSCs delays bone development in vivo

**Fig. 2:**
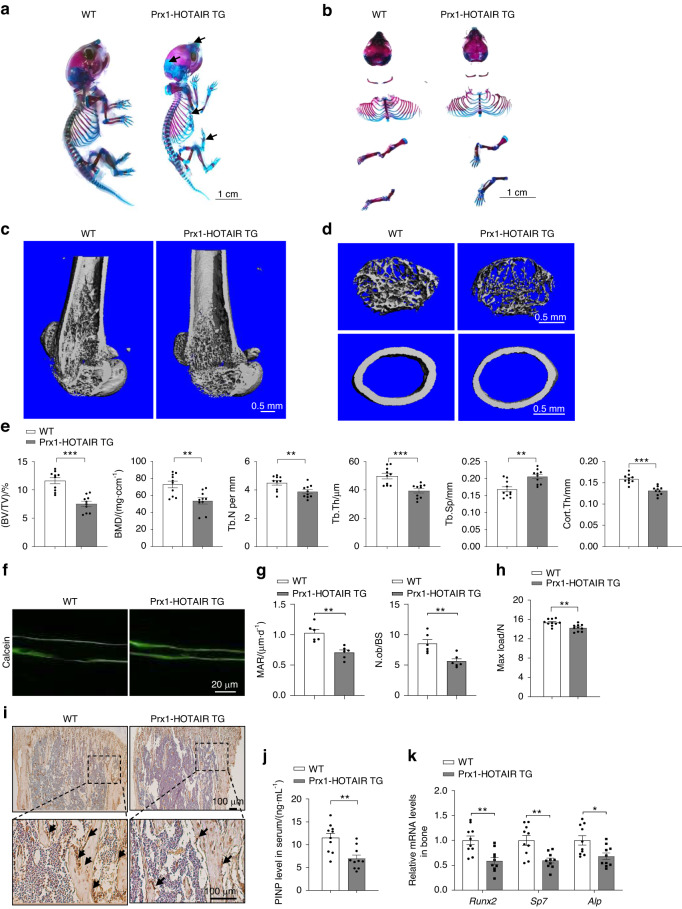
HOTAIR overexpression in MSCs delays bone development. **a**, **b** Alcian blue and Alizarin red staining of the whole skeletons of WT or Prx1-HOTAIR TG mice at 5 days old. Scale bar, 1 cm. **c** Representative images showing three-dimensional trabecular architecture by micro-CT reconstruction from WT and Prx1-HOTAIR TG male mice at 1 month old. Scale bar, 0.5 mm. **d** Representative images showing the three-dimensional trabecular architecture and cortical architecture as shown by micro-CT reconstruction at the distal femurs from WT and Prx1-HOTAIR TG mice at 1 month old. Scale bar, 0.5 mm. **e** Micro-CT measurements of BV/TV, BMD, Tb.N, Tb.Th, Tb.Sp, and Cort.Th in the distal femurs of mice. *n* = 10 for each group. BV/TV, ratio of bone volume to tissue volume; BMD, bone mineral density; Tb.N, trabecular number; Tb.Th, trabecular thickness; Tb.Sp, trabecular separation; Cort.Th, cortical bone thickness. **f** Representative images showing new bone formation assessed by double calcein labeling in WT and Prx1-HOTAIR TG mice. Scale bar, 20 μm. **g** Quantification of mineral apposition rate (MAR) and osteoblast number to bone surface (N.ob/BS) (*n* = 6 for each group). **h** The maximal (max.) load at failure determined by three-point bending of femurs from WT (*n* = 10) and Prx1-HOTAIR TG (*n* = 10) mice. **i** Histological images for Ocn staining of the proximal tibia from WT and Prx1-HOTAIR TG mice. Scale bar, 100 μm. **j** ELISA analysis of the PINP protein level in the serum from WT (*n* = 10) and Prx1-HOTAIR TG (*n* = 10) mice. **k** qRT-PCR analysis of *Alp*, *Runx2* and *Sp*7 mRNA levels in bone tissues collected from WT (*n* = 10) and Prx1-HOTAIR TG (*n* = 10) mice. Two-tailed unpaired Student’s *t* test was used for statistical evaluations of two group comparisons. All data are the mean ± s.e.m. *P < 0.05, **P < 0.01, ***P < 0.001

To explore the role of HOTAIR in BMSCs for bone development in vivo, we established transgenic mice with limb mesenchymal stem cell overexpression of HOTAIR (Prx1-HOTAIR TG) with a construct that expresses HOTAIR under the control of the *Prx1* promoter, which has been shown to be specifically activated in MSCs (Fig. S2a). The expression of HOTAIR was significantly higher in bone tissues from the Prx1-HOTAIR TG mice than in bone tissues from the WT mice but not in other tissues (Fig. S2b). We performed Alizarin red and Alcian Blue staining of whole skeletons from neonatal mice to evaluate bone development. The results showed that mineralization of skull and limb bones was obviously delayed in Prx1-HOTAIR TG mice compared with WT mice (Fig. 2a, b). At the age of 1 month, the Prx1-HOTAIR TG mice appeared smaller than the WT mice, and their body weight was also lower than that of the WT mice (Fig. S2c, d). Next, we analyzed the bone phonotype of the Prx1-HOTAIR TG and WT male mice at 1 month of age. Micro-CT analysis showed that Prx1-HOTAIR TG mice exhibited lower bone mass than WT mice in the femur and vertebra (Fig. 2c, d and Fig. S2e). In the femur, the bone volume per total volume (BV/TV), bone mineral density (BMD), trabecular number (Tb.N), trabecular thickness (Tb.Th) and cortical bone thickness (Cort.Th) of the Prx1-HOTAIR TG mice were significantly decreased compared with those of the WT mice, while the trabecular space (Tb.Sp) of the Prx1-HOTAIR TG mice was increased (Fig. 2e). Similar results were obtained in the vertebrae; BV/TV, BMD, Tb.N and Tb.Th were all significantly decreased, and Tb.Sp was accordingly increased in the Prx1-HOTAIR TG mice (Fig. S2f). Moreover, the mineral appositional rate (MAR) and osteoblast surface/bone surface (Ob.S/BS) were markedly reduced in the Prx1-HOTAIR TG mice (Fig. 2f, g). The strength of the long bones of the Prx1-HOTAIR TG mice was decreased (Fig. 2h). Immunostaining analysis revealed that the level of Ocn was obviously decreased in the bone tissues of the Prx1-HOTAIR TG mice (Fig. 2i). Accordingly, the level of the bone formation marker procollagen type I N-terminal propeptide (PINP) in serum from the Prx1-HOTAIR TG mice was also substantially decreased compared with that in the WT mice (Fig. 2j). Furthermore, the expression of osteogenic differentiation marker genes, including *Alp*, *Runx2*, and *Sp7*, was significantly decreased in bone tissues isolated from the Prx1-HOTAIR TG mice (Fig. 2k). However, body weight and bone mass showed no obvious changes between the WT and Prx1-HOTAIR TG mice at 6 months of age (Fig. S2g–j). These results demonstrated that HOTAIR overexpression in MSCs delays bone development.

### Osteoblast-specific HOTAIR overexpression promotes bone formation in vivo

**Fig. 3:**
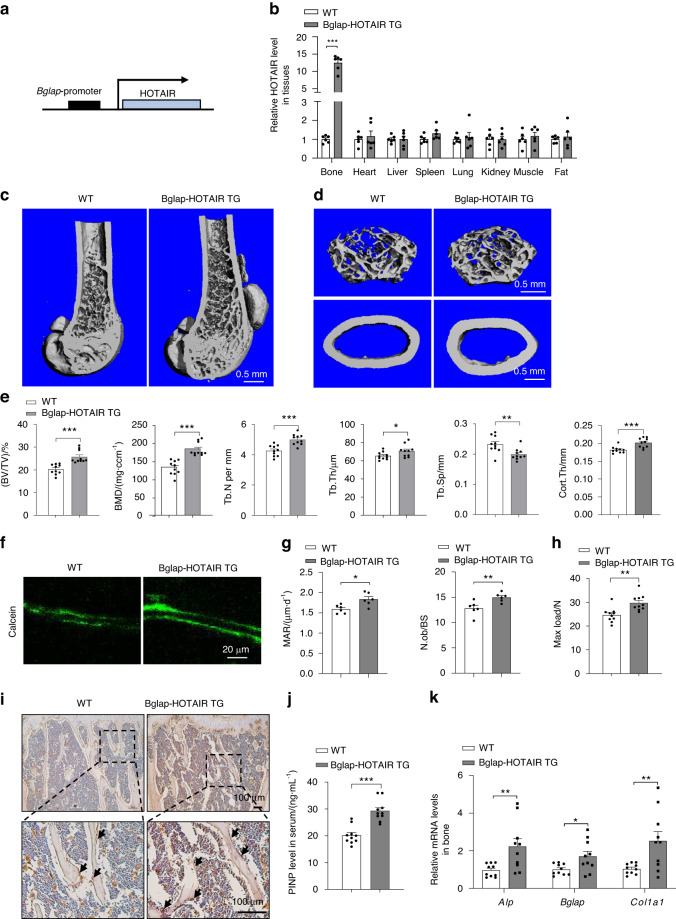
HOTAIR overexpression in osteoblasts increases bone formation. **a** Schematic representation of the transgenic construct used to generate osteoblast-specific HOTAIR overexpression transgenic mouse lines. **b** qRT‒PCR analysis of HOTAIR levels in bone and other tissues from 6-month-old WT and osteoblast-specific HOTAIR-overexpressing mice (Bglap-HOTAIR TG) (*n* = 6). **c** Representative images showing three-dimensional trabecular architecture by micro-CT reconstruction at 6 months of age. Scale bar, 0.5 mm. **d** Representative images showing the three-dimensional trabecular architecture and cortical architecture as shown by micro-CT reconstruction at the distal femurs at 6 months of age. Scale bar, 0.5 mm. **e** Micro-CT measurements of BV/TV, BMD, Tb.N, Tb.Th, Tb.Sp, and Cort.Th in the distal femurs of mice (*n* = 10 for each group). **f** Representative images showing new bone formation assessed by double calcein labeling. Scale bar, 20 μm. **g** Quantification of mineral apposition rate (MAR) and osteoblast number to bone surface (N.ob/BS) (*n* = 6 for each group). **h** The maximal (max.) load at failure determined by three-point bending of femurs from WT (*n* = 10) and Ocn-HOTAIR TG (*n* = 10) mice. **i** Histological images for Ocn staining of the proximal tibia. Scale bar, 100 μm. **j** ELISA analysis of the PINP protein level in the serum from WT (*n* = 10) and Bglap-HOTAIR TG (*n* = 10) mice. **k** qRT-PCR analysis of *Alp*, *Bglap* and *Col1a1* mRNA levels in bone tissues collected from WT (*n* = 10) and Bglap-HOTAIR TG (*n* = 10) mice. Two-tailed unpaired Student’s t test was used for statistical evaluations of two group comparisons. All data are the mean ± s.e.m. *P < 0.05, **P < 0.01, ***P < 0.001

To investigate the function of HOTAIR in osteoblast and bone formation in vivo, we developed a construct that expresses HOTAIR under the control of the *Bglap* promoter, which has been shown to be specifically activated in osteoblasts (Fig. 3a). Subsequently, we established Bglap-HOTAIR TG mice. The expression of HOTAIR was significantly higher in bone tissues from the Bglap-HOTAIR TG mice than in bone tissues from the WT mice but not in other tissues (Fig. 3b). At the age of 6 months, there were no obvious differences in body weight between the Bglap-HOTAIR TG and WT mice (Fig. S3a). Next, we performed micro-CT to analyze the bone phenotype of the femur and vertebra. The results showed that the Bglap-HOTAIR TG mice exhibited higher bone mass than the WT mice in the femur and vertebra (Fig. 3c, d). The bone structure indices, including BV/TV, BMD, Tb.N, Tb.Th and Cort.Th, were higher in the 6-month-old Bglap-HOTAIR TG mice than in the WT mice, while the Tb.Sp was lower in the femur (Fig. 3e). Similar results were obtained in the vertebra; BV/TV, Tb.N, Tb.Th and BMD were all significantly increased in the Bglap-HOTAIR mice (Fig. S3b, c). The results of calcein staining showed MAR and Ob.S/BS were markedly increased in the Bglap-HOTAIR TG mice (Fig. 3f, g). The strength of the long bones of the Bglap-HOTAIR TG mice was increased (Fig. 3h). The immunostaining analysis revealed that the level of the osteoblast marker Ocn was obviously increased in the tibias from the Bglap-HOTAIR TG mice (Fig. 3i). Accordingly, the level of PINP was significantly increased in serum from the Bglap-HOTAIR TG mice compared with WT mice (Fig. 3j). Furthermore, the mRNA levels of *Alp*, *osteocalcin* (*Bglap*) and *collagen 1* (*Col1a1*) in the bone tissue of the Bglap-HOTAIR TG mice were significantly increased (Fig. 3k). These results demonstrated that osteoblast-specific HOTAIR overexpression promotes bone formation.

### HOTAIR inhibits BMSC osteogenic differentiation and promotes osteoblast activity in vitro

**Fig. 4:**
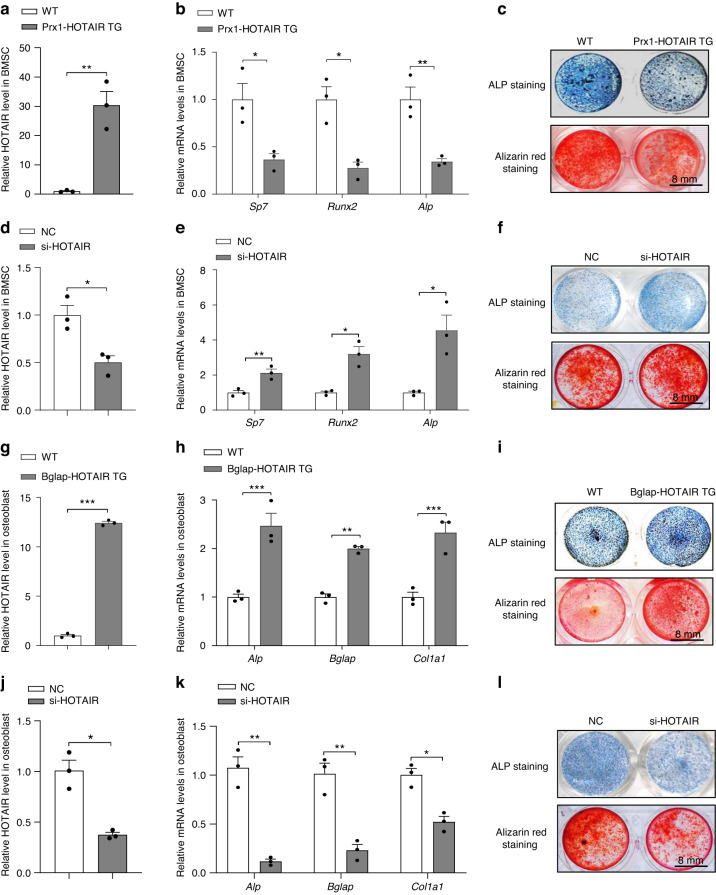
The expression and function of HOTAIR in BMSCs and osteoblasts. **a** qRT‒PCR analysis of HOTAIR levels in BMSCs isolated from WT and Prx-HOTAIR TG mice. **b** qRT‒PCR analysis of *Sp7*, *Runx2*, and *Alp* mRNA levels in BMSCs isolated from WT and Prx-HOTAIR TG mice. **c** Representative images of ALP staining (top) in BMSCs cultured with osteogenic medium for 3 days and Alizarin red staining (bottom) in BMSCs cultured with osteogenic medium for 21 days. Scale bar, 8 mm. **d** qRT‒PCR analysis of HOTAIR levels in BMSCs cultured with osteogenic medium for 3 days. **e** qRT‒PCR analysis of *Sp7*, *Runx2*, and *Alp* mRNA levels in BMSCs cultured with osteogenic medium for 3 days. **f** Representative images of ALP staining in BMSCs cultured with osteogenic medium for 3 days and Alizarin red staining (below) in BMSCs cultured with osteogenic medium for 21 days. Scale bar, 8 mm. **g** qRT‒PCR analysis of HOTAIR levels in primary osteoblasts isolated from WT and Bglap-HOTAIR TG mice and cultured with osteogenic medium for 3 days. **h** qRT‒PCR analysis of *Alp*, *Bglap*, and *Col1a1* mRNA levels in primary osteoblasts cultured with osteogenic medium for 3 days. **i** Representative images of ALP staining (top) in primary osteoblasts cultured with osteogenic medium for 3 days. Alizarin red staining (below) in primary osteoblasts cultured with osteogenic medium for 14 days. **j** qRT‒PCR analysis of HOTAIR levels in primary osteoblasts cultured with osteogenic medium for 3 days. **k** qRT‒PCR analysis of *Alp*, *Bglap*, and *Col1a1* mRNA levels in primary osteoblasts cultured with osteogenic medium for 3 days. **l** Representative images of ALP staining (top) in primary osteoblasts cultured with osteogenic medium for 3 days. Alizarin red staining (below) in primary osteoblasts cultured with osteogenic medium for 14 days. Scale bar, 8 mm. All data are the mean ± s.e.m. from three independent experiments. Two-tailed unpaired Student’s *t* test was used for statistical evaluations of two group comparisons. *P < 0.05, **P < 0.01, ***P < 0.001

To investigate the effect of HOTAIR on BMSCs, we isolated BMSCs from Prx1-HOTAIR TG mice and WT mice. First, we detected the level of HOTAIR in BMSCs, and HOTAIR TG BMSCs showed significantly higher levels of HOTAIR than BMSCs (Fig. 4a). Accordingly, the HOTAIR TG BMSCs showed decreased expression of the BMSC marker genes *Sp7*, *Runx2* and *Alp* (Fig. 4b). ALP staining and Alizarin red staining showed that ALP activity and mineralization capacity was reduced in the HOTAIR TG BMSCs compared with the WT BMSCs (Fig. 4c). Next, we used siRNA to knock down HOTAIR in BMSCs. The results showed that the expression of *Sp7*, *Runx2*, and *Alp* was increased during the osteogenic differentiation of BMSCs, and ALP activity and mineralization capacity were also enhanced (Fig. 4d–f). To investigate the role of HOTAIR in osteoblasts, we isolated osteoblasts from the Bglap-HOTAIR TG mice and the WT mice. The results showed that the level of HOTAIR in the Bglap-HOTAIR TG osteoblasts was increased approximately 12.4-fold compared with that in the WT osteoblasts (Fig. 4g). Furthermore, we found that the expression of *Alp*, *Bglap*, and *Col1a1* in the Bglap-HOTAIR TG osteoblasts was increased (Fig. 4h). Correspondingly, ALP activity and mineralization capacity were enhanced in the Bglap-HOTAIR TG osteoblasts (Fig. 4i). In contrast, HOTAIR siRNA in osteoblasts significantly inhibited the expression of *Alp*, *Bglap*, and *Col1a1* (Fig. 4j, k), while ALP staining and Alizarin red staining were also obviously decreased (Fig. 4l). These data indicated that HOTAIR inhibits the osteogenic differentiation of BMSCs but promotes osteoblast activity.

### HuR mediates the nucleocytoplasmic translocation of HOTAIR during osteogenic differentiation of BMSCs

**Fig. 5:**
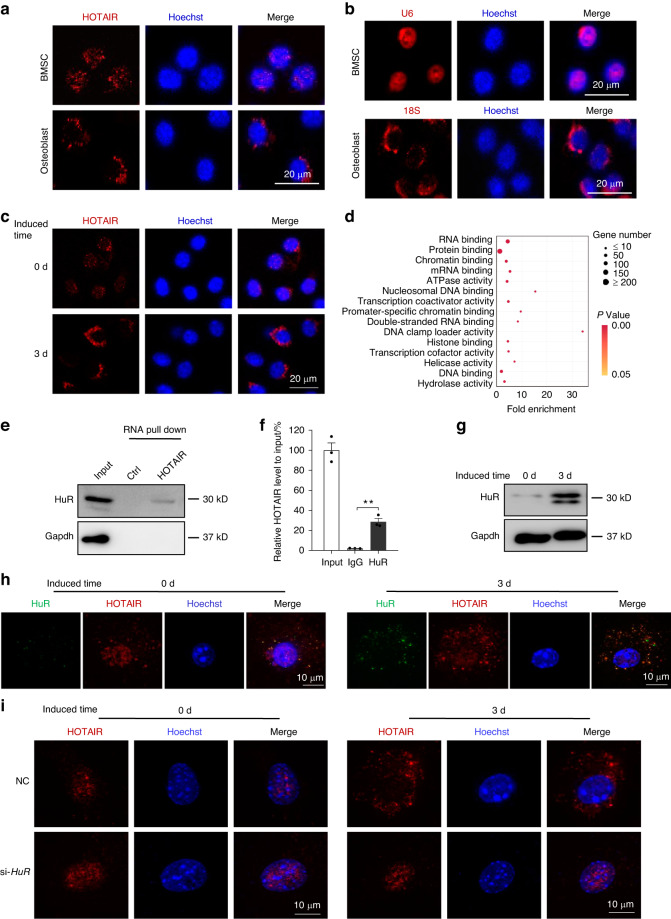
HuR is essential for the nucleocytoplasmic translocation of HOTAIR. **a** RNA-fluorescent in situ hybridization (FISH) was conducted to detect HOTAIR localization using digoxigenin (DIG) labeling probes specific for HOTAIR sequences (red). Nuclei were stained with Hoechst (blue). Scale bar, 20 μm. **b** Detection of U6 and 18 S RNA using RNA probes labeled with Cy3 (red). Nuclei were stained with Hoechst (blue). Scale bar, 20 μm. **c** RNA FISH to analyze the HOTAIR localization in BMSCs cultured with osteogenic medium for 0 days and 3 days. Scale bar, 20 μm. **d** KEGG analysis of the HOTAIR binding proteins in BMSCs through biotin-labeled full-length HOTAIR or biotin-labeled control RNA-based RNA pulldown by mass spectrometry. **e** Western blot analysis of HOTAIR binding protein HuR in BMSCs by biotinylated RNA pulldown. **f** qRT‒PCR analysis of HOTAIR levels by RNA immunoprecipitation in BMSCs with a HuR antibody, and rabbit IgG was used as a control. **g** Western blot analysis of HuR protein levels in BMSCs cultured with osteogenic medium for 0 days and 3 days. **h** Structured illumination microscopy (SIM) images for the colocalization of HuR and HOTAIR in BMSCs cultured with osteogenic medium for 0 days and 3 days. FISH was conducted to detect HOTAIR using DIG labeling probes specific for HOTAIR sequences and TRITC secondary antibody (red). Immunofluorescence was performed to detect HuR using a HuR antibody and FITC secondary antibody (green). Scale bar, 10 μm. **i** The distribution of HOTAIR in BMSCs cultured with osteogenic medium for 0 days and 3 days. Scale bar, 10 μm. All data are the mean ± s.e.m. from three independent experiments. Two-tailed unpaired Student’s t test was used for statistical evaluations of two group comparisons. **P < 0.01

The function of HOTAIR in BMSCs and osteoblasts is dependent on its specific subcellular localization. To detect the localization of HOTAIR in BMSCs and osteoblasts, we performed RNA fluorescence in situ hybridization (FISH) in BMSCs and osteoblasts with a digoxigenin-labeled HOTAIR probe. Interestingly, we found that HOTAIR was mainly located in the nucleus of BMSCs and primarily in the cytoplasm of osteoblasts (Fig. 5a). U6 and 18 S rRNA were used as positive controls for the nuclear and cytoplasmic fractions, respectively (Fig. 5b). To examine whether HOTAIR translocates from the nucleus to the cytoplasm during the osteogenic differentiation of BMSCs, we performed RNA FISH to analyze the changes in HOTAIR localization. The results showed that HOTAIR was mainly located in the nucleus in BMSCs, and thereafter, HOTAIR was transported to the cytoplasm when cells were cultured in osteogenic induction medium for 3 days (Fig. 5c). To elucidate the mechanism underlying the translocation of HOTAIR from the nucleus to the cytoplasm during osteogenic differentiation of BMSCs, we performed RIP by biotinylated full-length HOTAIR and mass spectrometry to analyze the HOTAIR binding proteins in BMSCs. The results showed that 321 proteins were specifically identified in the biotinylated HOTAIR IP group (Table S1). According to the KEGG enrichment analysis, several types of important biological processes, including RNA binding, chromatin binding, ATPase activity, and transcription coactivator activity, may participate in RNA translocation (Fig. 5d). Among them, HuR was one of the top enriched RNA binding proteins and was reported to be capable of translocation between the nucleus and cytoplasm. To verify the interaction between HOTAIR and HuR, we performed a biotinylated RNA pulldown assay. The results showed that HOTAIR bound to HuR (Fig. 5e). We also carried out RIP using a HuR antibody in BMSCs, and the results showed that HOTAIR was significantly enriched in the HuR antibody group compared with the IgG group (Fig. 5f). Moreover, we found that HuR protein levels were significantly increased with the osteogenic differentiation of BMSCs (Fig. 5g). To demonstrate that both HOTAIR and HuR translocated from the nucleus to the cytoplasm during osteoblastic differentiation, we conducted FISH and immunofluorescence assays to detect the levels and locations of HOTAIR and HuR on Day 0 and Day 3. The results showed that HuR was expressed at low levels on Day 0, while HOTAIR was primarily located in the nucleus. Then, an increase in HuR expression and colocalization with HOTAIR was observed on Day 3, as evidenced by SIM microscopy (Fig. 5h). Furthermore, *HuR* knockdown inhibited the translocation of HOTAIR from the nucleus to the cytoplasm during osteogenic differentiation of BMSCs (Fig. 5i). These results indicated that HuR mediates the nucleocytoplasmic translocation of HOTAIR during the osteogenic differentiation of BMSCs.

### HuR plays an essential role in the regulation of HOTAIR on osteoblast function in the cytoplasm

**Fig. 6:**
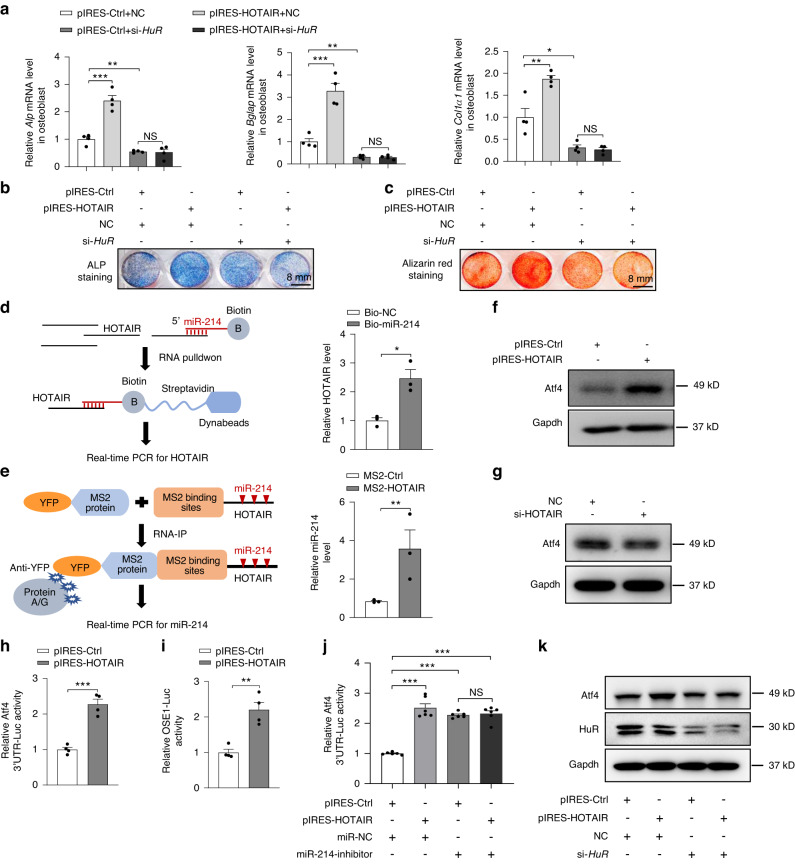
HuR mediated the effect of HOTAIR on osteoblast function by sponging miR-214 in the cytoplasm. **a** qRT‒PCR analysis of *Alp*, *Bglap*, and *Col1a1* mRNA levels in primary osteoblasts transfected with pIRES-HOTAIR and pIRES-Ctrl with or without *HuR* knockdown and cultured with osteogenic medium for 3 days. **b**, **c** Representative images of ALP staining (**b**) in primary osteoblasts cultured with osteogenic medium for 3 days and Alizarin red staining (**c**) in primary osteoblasts cultured with osteogenic medium for 14 days. Scale bar, 8 mm. **d** Analysis of the interaction of HOTAIR and miR-214 by biotin-labeled miR-214 probe-based RNA pulldown assays. Left, schematic diagram of the miR-214-based RNA pulldown assay. Right, qRT‒PCR was performed with RIP samples from osteoblasts using biotin-labeled miR-214 probes or biotin-labeled negative control (NC). **e** Analysis of the interaction of HOTAIR and miR-214 by MS2-based pulldown assay. Left, schematic diagram of the MS2-based RNA immunoprecipitation (RIP) assay. Right, qRT‒PCR was performed with RIP samples from osteoblasts transfected with pcDNA3.1-12 x MS2 (control vector), pcDNA3.1-HOTAIR-12xMS2, or YFP-MS2. **f** Western blot analysis of Atf4 protein levels in osteoblasts transfected with pIRES-HOTAIR or pIRES-Ctrl. **g** Western blot analysis of Atf4 protein levels in osteoblasts transfected with si-HOTAIR and NC. **h** The luciferase activity of Atf4 3’UTR in osteoblasts transfected with pIRES-HOTAIR or pIRES-Ctrl. **i** The luciferase activity of OSE1 in osteoblasts transfected with pIRES-HOTAIR or pIRES-Ctrl. **j** The luciferase activity of Atf4 3’UTR in osteoblasts transfected with pIRES-HOTAIR or pIRES-Ctrl with or without miR-214 inhibition. **k** Western blot analysis of Atf4 and HuR protein levels in primary osteoblasts transfected with pIRES-HOTAIR and pIRES-Ctrl with or without *HuR* knockdown and cultured with osteogenic medium for 3 days. All data are the mean ± s.e.m. from three independent experiments. Statistical analysis with more than two groups was performed with one-way analysis of variance (ANOVA) with Tukey’s multiple comparison test to determine group differences. *P < 0.05, **P < 0.01, ***P < 0.001. NS, not significant

To investigate the essential role of HuR in the regulation of osteoblast function by HOTAIR, we evaluated the effect of HOTAIR overexpression on osteoblast function in the presence or absence of *HuR*. The results showed that *HuR* knockdown remarkably abrogated the enhancement of osteoblast function by HOTAIR (Fig. 6a–c). Cytoplasmic lncRNAs can act as competing endogenous RNAs (ceRNAs), and sponge miRNAs, and facilitate the protein expression of miRNA target genes. We predicted the miRNAs that can interact with HOTAIR and found that HOTAIR had nine binding sites for miR-214-3p (Fig. S4). It has also been reported that HOTAIR promotes proliferation and inhibits apoptosis by sponging miR-214-3p in cancer cells.^34^ To verify the interaction between HOTAIR and miR-214, we carried out RNA RIP with biotin-labeled miR-214. The results showed that HOTAIR was significantly enriched in the biotin-labeled miR-214 group compared with the biotin-labeled negative control group (Fig. 6d). Moreover, we performed another RNA IP assay with an anti-YFP antibody to determine whether miR-214 could be pulled down by YFP-MS2-tagged HOTAIR. MiR-214 was significantly enriched by the YFP antibody compared with the nonspecific IgG control antibody (Fig. 6e). Furthermore, HOTAIR overexpression significantly increased the protein level of Atf4, whereas knockdown of HOTAIR decreased the protein level of Atf4 in osteoblasts (Fig. 6f, g). Moreover, HOTAIR overexpression significantly increased the luciferase activities of *Atf4* 3’UTR and OSE1 in osteoblasts (Fig. 6h, i). When miR-214 was inhibited, the activity of *the Atf4* 3’UTR could not be activated by HOTAIR (Fig. 6j). In addition, *HuR* knockdown abrogated the enhancement of Atf4 protein levels by HOTAIR (Fig. 6k). These results indicated that HOTAIR increases the Atf4 protein level by sponging miR-214 in osteoblasts. HOTAIR has been reported to be a scaffold of the PRC2 complex and represses gene expression by increasing histone methylation.^20,21^ Our results showed that HOTAIR bound to Ezh2 in BMSCs (Fig. S5a). Moreover, RNA immunoprecipitation (RIP) and qRT‒PCR results showed that HOTAIR was significantly enriched in the Ezh2-specific antibody treatment group compared with the IgG group (Fig. S5b). Furthermore, the global H3K27me3 level was significantly decreased in the BMSCs transfected with HOTAIR siRNA compared with the control cells (Fig. S5c). Then, we used a chromatin immunoprecipitation (ChIP) assay and qRT‒PCR to analyze the effect of HOTAIR on H3K27me3 modification in the promoter regions of the *Runx2* and *Sp7* genes. The results showed that the H3K27me3-rich *Runx2* and *Sp7* promoter regions were significantly decreased in BMSCs after the knockdown of HOTAIR (Fig. S5d, e). Moreover, Ezh2 knockdown attenuated the effect of HOTAIR on H3K27me3 levels in BMSCs (Fig. S5f), which indicated that HOTAIR regulates H3K27me3 levels through Ezh2. These results indicated that nuclear HOTAIR promotes H3K27me3 modifications of Runx2 and Sp7 promoter regions in BMSCs.

### Osteoblast-specific HOTAIR overexpression attenuates bone loss induced by unloading

**Fig. 7:**
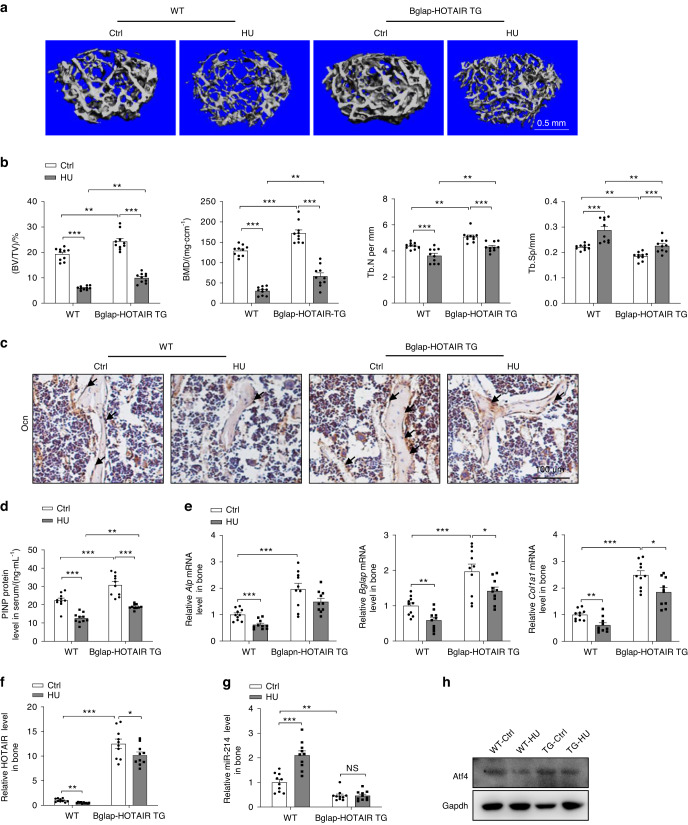
Osteoblast-specific overexpression of HOTAIR alleviates unloading-induced bone loss. **a** Representative image showing three-dimensional distal femur trabecular architecture by micro-CT reconstruction from the indicated groups of mice. Representative images of six independent tissues in each group. Ctrl, control group, HU, hindlimb unloading group. Scale bar, 0.5 mm. **b** Micro-CT measurements for BV/TV, BMD, Tb.N and Tb.Sp at the distal femurs. *n* = 10 for each group. **c** Representative images of Ocn staining of the proximal tibia. Scale bar, 100 μm. **d** ELISA analysis of PINP protein levels in the serum. *n* = 10 for each group. **e** qRT-PCR analysis of *Alp*, *Bglap*, and *Col1a1* mRNA levels in bone tissues. *n* = 10 for each group. **f**, **g** qRT-PCR analysis of HOTAIR and miR-214 levels in bone tissues. *n* = 10 for each group. **h** Western blot analysis of Atf4 protein levels in bone tissue. All data are the mean ± s.e.m. Statistical analysis with more than two groups was performed with two-way analysis of variance (ANOVA) with the Šídák post hoc test to determine group differences. All data are the mean ± s.e.m. *P < 0.05, **P < 0.01, ***P < 0.001

To evaluate the therapeutic potential role of HOTAIR in bone loss, we employed the commonly used hindlimb unloading (HU) model to examine the bone remodeling process of WT, Prx1-HOTAIR TG, and Bglap-HOTAIR TG male mice at the age of 6 months. When the 6-month-old mice were subjected to 28 days of HU, the trabecular bone mass and architecture-related parameters, including BMD, BV/TV, Tb.N and Tb.Th, were significantly reduced, and SMI and Tb.Sp were increased in both WT and Prx1-HOTAIR TG mice; however, the indices showed no obvious difference between the WT and Prx1-HOTAIR TG mice (Fig. S6a, b). In contrast, the trabecular bone mass and architecture-related parameters, including BMD, BV/TV, and Tb.N, were significantly increased, and Tb.Sp was markedly reduced in the Bglap-HOTAIR TG mice compared with the WT mice after HU (Fig. 7a, b). Furthermore, the Ocn level in bone tissues was obviously higher in the Bglap-HOTAIR TG mice under HU conditions than in the WT mice (Fig. 7c). Accordingly, serum PINP levels were also significantly elevated in the Bglap-HOTAIR TG mice after HU (Fig. 7d). Moreover, the decrease in osteoblast marker gene expression induced by HU was alleviated in the Bglap-HOTAIR TG mice (Fig. 7e). To confirm the mechanism of HOTAIR sponging miR-214 in the HU model, we detected changes in HOTAIR and miR-214 levels and Atf4 protein in the bone tissue of hindlimb unloading. The results showed that the level of HOTAIR was decreased in the bone of the WT mice after HU treatment, the miR-214 level was increased, and the Atf4 protein level was decreased accordingly (Fig. 7f, g). The Bglap-HOTAIR TG mice showed an alleviated reduction in Atf4 protein in the bone tissue of mice after treatment with HU (Fig. 7h). These results demonstrated that osteoblast-specific HOTAIR overexpression attenuated HU-induced bone loss.

## Discussion

**Fig. 8:**
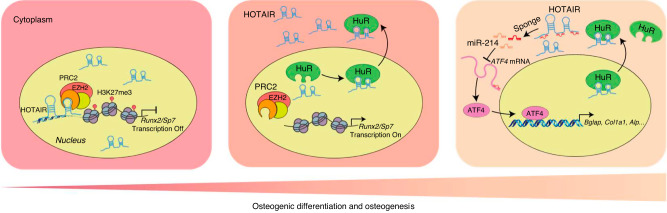
Model of the distinct function of HOTAIR in BMSC osteogenic differentiation and osteogenesis. In BMSCs, nuclear HOTAIR interacts with EZH2 and enhances the modification of H3K27me3 in the promoter regions of *Runx2* and *Sp7* genes, which leads to the inhibition of osteogenic differentiation of BMSCs. In osteoblasts, HOTAIR is located in the cytoplasm to sponge miR-214 and promotes osteoblast function by alleviating Atf4 translation. Moreover, HuR mediates the nucleocytoplasmic translocation of HOTAIR in the progression of osteogenic differentiation of BMSCs

In this study, we found that nucleocytoplasmic translocation of HOTAIR plays an important role in BMSC osteogenic differentiation and osteogenesis (Fig. 8). We first identified that Prx1-HOTAIR transgenic mice exhibited delayed bone development, while osteoblast-specific Bglap-HOTAIR transgenic mice presented higher bone mass and showed inhibition of unloading-induced bone loss in vivo. The lncRNA HOTAIR acted as a novel regulator of BMSC osteogenic differentiation and osteogenesis, which was dependent on its specific subcellular localization. In BMSCs, HOTAIR colocalized with HuR, which was essential for the translocation of HOTAIR from the nucleus to the cytoplasm during osteogenesis. Cytoplasmic HOTAIR in osteoblasts sequestered miR-214 and promoted osteoblast function. This study offers insight into the exact regulation of HOTAIR during different stages of bone formation. Additionally, we proposed a new paradigm for the mechanism regulating lncRNA subcellular localization.

LncRNAs are long noncoding RNAs that can regulate various biological processes, and there are more than 16 000 lncRNAs in humans.^6^ The expression of lncRNAs occurs in a cell-type and tissue-specific manner.^35,36^ Increasing evidence has demonstrated that lncRNAs play important roles in osteogenic differentiation of BMSCs and bone formation, which is subjected to precise regulation. HOTAIR is one of the most extensively studied lncRNAs found in human cancer.^37^ It was reported that knockout of HOTAIR led to the homeotic transformation of the spine and malformation of metacarpal bone,^27^ which suggests that the effect of HOTAIR is closely related to bone development. Moreover, the bone phenotype of HOTAIR knockout mice remains controversial in different research teams.^33^ Several studies have also reported that HOTAIR is involved in regulating the process of human BMSC osteogenic differentiation and osteogenesis. However, the specific role of HOTAIR in the osteogenic differentiation of BMSCs and bone formation has not been fully revealed, and a gain-of-function model is needed. Our results demonstrated that HOTAIR is a key regulator of the osteogenic differentiation of BMSCs and osteogenesis dependent on its distinct subcellular localization. Interestingly, Prx1-HOTAIR TG mice showed a delayed bone mineralization phenotype, whereas osteoblast-specific HOTAIR overexpression increased bone formation and prevented bone loss induced by hindlimb unloading. Our study first reported that the same lncRNA had distinct functions in different stages of bone formation, which conferred different bone phenotypes.

The diverse functions of lncRNAs are strictly related to their unique subcellular localization.^38,39^ Nuclear lncRNAs participate in gene regulation at the epigenetic and transcriptional levels, including histone modifications, the regulation of DNA methylation and chromatin remodeling, and interactions with chromatin modification complexes, transcription factors, and proteins in the nucleus.^12,40,41^ Moreover, lncRNAs in the cytoplasm participate in gene regulation at the post-transcriptional and translational levels or protein modification and stability, including interaction with proteins in the cytoplasm and the regulation of mRNA metabolism, as competitive endogenous RNAs (ceRNAs) interact with microRNAs.^22,38,40,42^

Although the function of lncRNAs is diverse according to intracellular distribution, the nucleocytoplasmic translocation of lncRNAs has rarely been explored. In the nucleus, HOTAIR can recruit PRC2 and other chromatin regulators to repress transcription of the HOX D locus.^21^ HOTAIR also adjusts the cancer epigenome by regulating histone H3K27me3 by targeting PRC2.^20^ HOTAIR can bind to many genomic sites through its GA-rich motif in the nucleus.^43^ Moreover, in the cytoplasm, HOTAIR binds to the ubiquitin ligases E3, Dzip3, and Mex3b to promote ubiquitination and proteolysis of Ataxin-1 and snurportin-1 in senescent cells.^42^ HOTAIR can also function as a miRNA sponge in the cytoplasm.^44^ However, studies have reported that HOTAIR acts as a ceRNA to sponge miR-214-3p in hepatocellular carcinoma progression,^45^ and miR-214-3p targets ATF4 to inhibit osteoblast activities and bone formation.^46^ However, the intracellular distribution of HOTAIR in BMSCs and osteoblasts is unknown. We found that HOTAIR was mainly located in the nucleus of BMSCs but was primarily located in the cytoplasm of osteoblasts. Moreover, HOTAIR can translocate from the nucleus to the cytoplasm during the osteogenic differentiation of BMSCs. HOTAIR in the nucleus inhibited the osteogenic differentiation of BMSCs, while HOTAIR in the cytoplasm enhanced osteoblast function by sponging miR-214. The different subcellular localization of HOTAIR in BMSCs and osteoblasts demonstrates its distinct function in bone formation.

Although the roles of lncRNAs are diverse, the mechanisms of their intracellular localization are rarely explored. RNA-binding proteins (RBPs) are a class of specific proteins that bind to RNA.^47^ RBPs interact with lncRNAs through one or multiple globular RNA-binding domains (RBDs) and regulate the fate or function of lncRNAs.^13^ RBPs can affect RNA stability, localization, and processing.^48,49^ Therefore, identifying the role of RBPs is important for understanding the mechanisms of lncRNA intracellular localization. HuR is a universally expressed RBP.^50^ This molecule recognizes target mRNAs by binding to AU-rich elements in the 3′UTR and enhances mRNA stability and translation.^51^ HuR was reported to be crucial for the nuclear import of cellular retinoic acid-binding protein 2 (CRABP2)^52^ and acts as a negative regulator in adipogenesis^53^ and promotes bone regeneration by interacting with circStag1.^54^ HuR has been reported to promote bladder cancer progression by competitively binding to HOTAIR with miR-1,^55^ and the feed-forward regulatory loop between HuR and HOTAIR promotes head and neck squamous cell carcinoma progression and metastasis.^56^ Downregulating HuR and IGF2BP1 by suppressing lncRNA HOTAIR results in inhibited growth of breast cancer cells.^57^ However, it remains unclear whether HuR can regulate the intracellular localization of lncRNAs. In this study, we verified that HuR acts as a transporter for HOTAIR nucleocytoplasmic translocation. HuR specifically interacts with HOTAIR in the nucleus of BMSCs, and knockdown of HuR leads to HOTAIR nuclear retention and inhibits the osteogenic differentiation of BMSCs. HuR plays an essential role in HOTAIR nucleocytoplasmic translocation.

The mouse model of hindlimb unloading (HU) has been proven to mimic the loss of mechanical loading experienced by astronauts during spaceflight and is accepted as a method for disuse-induced bone loss.^58,59^ Many studies using the HU model have highlighted a more significant loss in both bone mass and architecture of the trabecular bone.^60^ Although treatment strategies such as drugs and genetically modified mice have been widely used to evaluate the therapeutic effect of this model, the mechanisms of bone loss caused by this model remain unclear. Our results proved the distinct effect of Bglap-HOTAIR and Prx1-HOTAIR mice on hindlimb unloading-induced bone loss, which provided a new vision and strategies for bone loss prevention. The conservation of HOTAIR sequences is not very high; however, HOTAIR has considerably conserved structures in mammals.^61^ The conserved structures of HOTAIR may have an important regulatory function in both humans and mice.

Taken together, our studies not only revealed that the nucleocytoplasmic translocation of HOTAIR is critical for BMSC osteogenic differentiation and bone formation in vitro and in vivo but also demonstrated that HuR colocalizes with HOTAIR and mediates its nucleocytoplasmic translocation during the progression of osteogenic differentiation of BMSCs. We first reported the distinct role of HOTAIR in the osteogenic differentiation of BMSCs depending on their cellular localization and revealed its mechanism of nucleocytoplasmic translocation mediated by HuR. These findings provide precise regulation of lncRNAs in temporal and spatial patterns during the osteogenic differentiation of BMSCs and offer a therapeutic strategy for osteoporosis.

## Materials And Methods

### Mice

The plasmid pOG2-Cre (a gift from Xiao Yang, National Center for Protein Sciences, Beijing, China), which contains a 0.9 kb Bglap promoter to drive osteoblast-specific gene expression, was used to generate Bglap-HOTAIR transgenic mice (Bglap-HOTAIR TG). The plasmid pBS-mPrx1 (#13961, Addgene), which contains the 2.4 kb Prx1 promoter to drive limb mesenchymal stem cell-specific gene expression, was used to generate Prx1-HOTAIR transgenic mice (Prx1-HOTAIR TG). The Bglap-HOTAIR plasmid was constructed by inserting HOTAIR into the corresponding site downstream of the Bglap promoter. The Prx1-HOTAIR plasmid was constructed by inserting HOTAIR into the corresponding site downstream of the Prx1 promoter. These plasmids were linearized with KpnI, and then, the fragments of Bglap-HOTAIR/Prx1-HOTAIR were purified and microinjected into C57BL/6 J mouse embryos. The embryos were then surgically transferred into pseudopregnant B6D2F1 (C57BL/6 X DBA2) female mice as foster mothers at the Laboratory Animal Research Center of Tsinghua University. Animals were bred and maintained under specific pathogen-free (SPF) conditions in the Animal Research Building of the China Astronaut Research and Training Center (12 h light, 12 h dark cycles, temperature controlled at 21 ± 2 °C with free access to food and water). All the experimental procedures were approved by the Committees of Animal Ethics and Experimental Safety of the China Astronaut Research and Training Center (Reference number: ACC-IACUC-2020–006).

### Immunohistochemistry and bone histomorphometry

The tibiae from mice were fixed with 4% PFA for 48 h, decalcified in 10% EDTA solution for 2 weeks, and then embedded in paraffin. The bone tissue was cut into approximately 5–7 μm thin slices by a microtome and absorbed on the slide. According to the IHC protocol, paraffin-embedded sections were dewaxed in xylene and rehydrated in gradient ethanol. Protease K was used for antigen retrieval at room temperature or 37 °C for 30 min, and then, a solution of 3.0% H_2_O_2_ was used to block the activity of endogenous peroxidase. The sections were blocked with goat serum and then incubated with an antibody against osteocalcin (1:500, Proteintech, 23418-1-AP) overnight at 4 °C. After three washes with PBS, horseradish peroxidase-labeled secondary antibodies were added and incubated at room temperature for 1 h, and then color development was performed with a DAB kit (ZSGB-bio). The sections were examined using a microscope (ECLIPSE CiS, Nikon).

### Micro-CT

The distal femurs, cortical bone, and fifth lumbar vertebra bone of mice were scanned by a micro-CT system (SCANCO Medical μ40, Bruttisellen, Switzerland). For distal femurs, the region of trabecular bone proximal to the distal growth plate was selected for analyses within a conforming volume of interest commencing at a distance of 210 μm from the growth plate and extending a further longitudinal distance of 1 050 μm in the proximal direction. Cortical bone measurements were performed in the diaphyseal region of the femur starting at a distance of approximately 3.50 mm from the growth plate and extending a further longitudinal distance of 840 μm in the proximal direction. The trabecular region of the fifth lumbar vertebra bone was defined manually to exclude the cortical component. All trabecular bone from each selected slice was segmented for 3-D reconstruction to calculate the following parameters: bone volume per total volume (BV/TV), bone mineral density (BMD), trabecular number (Tb.N), trabecular thickness (Tb.Th), trabecular space (Tb.Sp) and structure model index (SMI).

### OVX mouse model

All the female mice used were maintained under standard animal housing conditions. The 3-month-old female mice were divided into ovariectomy (OVX) or sham-operated groups. At 3 months after OVX surgery, bone tissues and bone marrow cells were collected from sham-operated and OVX mice. All of the experimental procedures were approved by the Committees of Animal Ethics and Experimental Safety of the China Astronaut Research and Training Center.

### Aged mouse model

Twenty-four-month-old male mice were purchased from Yangzhou Youdu Biotechnology Co., Ltd., and 2-month-old male mice were used as controls for young mice. Bone tissues and bone marrow cells were collected from young and aged mice. All of the experimental procedures were approved by the Committees of Animal Ethics and Experimental Safety of the China Astronaut Research and Training Center.

### Three-point bending analysis

The femurs were stored in phosphate buffer immediately after being removed from the mice. The three-point bending test (span length, 4.0 mm; loading speed 0.50 mm·s^−1^) at the mid-femur was performed using Texture Analyzer Texture Pro CT V1.6 Build, Brookfield Engineering Labs, Inc.

### Hindlimb unloading (HU) mouse model

Hindlimb unloading was performed to remove body weight-induced mechanical loading on hindlimbs, which mimicked the bone loss induced by weightlessness.^62^ The mice were individually caged and suspended by the tail using a strip of adhesive surgical tape attached to a chain hanging from a pulley. The mice were suspended at a 30° angle to the floor with only the forelimbs touching the floor; this allowed the mice to move and access food and water freely. The 3-month-old male mice were subjected to hindlimb unloading with tail suspension for 28 d. After euthanasia, bilateral femurs and tibiae were dissected and processed for cell sorting with a flow cytometer, micro-CT examination, bone histomorphometry analysis, and qPCR analysis.

### Fluorescence activated cell sorting (FACS)

Whole bone marrow cells were flushed with α-MEM containing 10% penicillin‒streptomycin from the bone tissue of hindlimb unloading (HU, 3-month-old male mice were subjected to hindlimb unloading with tail suspension for 28 d), ovariectomy (OVX, 3-month-old female mice were OVX or sham-operated for 3 months) and aging model (24-month-old male mice) mice. Flow cytometry was used to isolate Sca-1^+^CD29^+^CD45^−^CD11b^−^ BMSCs and ALP^+^ osteoblasts. Cells were sorted in TRIzol reagent for RNA extraction and qPCR analysis, and specific primers are listed in Table S2.

### Measurement of serum PINP concentrations

The serum levels of PINP were detected by a mouse PINP (procollagen type I N-terminal propeptide) ELISA kit (ImmunoWay, KE1744) according to the product instructions.

### Alkaline phosphatase staining and Alizarin red staining

For alkaline phosphatase staining, the cells were fixed with 4% paraformaldehyde (PFA) for 10 min and rinsed with PBS three times at room temperature. Alkaline phosphatase staining was monitored using a Vector Blue Substrate Kit (SK-5300; Vector Laboratories). According to the protocol, the cells were incubated with the substrate working solution for 20–30 min and then rinsed with PBS. For Alizarin red staining, cells were cultured in an osteogenic induction medium for 14 days, fixed with 4% PFA and stained with 40 mmol·L^−1^ Alizarin red S (ARS, Sigma-Aldrich, A-5533) at a pH of 4.0 for 15 min with gentle agitation. Then, the cells were rinsed five times with double-distilled H_2_O while gently agitating. Both staining procedures were protected from light.

### Double calcein labeling

Peritoneal injection with calcein (30 mg·kg^−1^ body weight) was performed at 12 and 2 days before mouse euthanasia. The tibias were harvested for undecalcified histology analysis. Unstained 15 mm sections were examined using fluorescence microscopy. Statistical analyses were performed with the BioquantOsteo Analysis System.

### Whole-mount staining

Skeletal preparations were stained with ARS and Alcian blue as previously described.^63^ In brief, newborns were eviscerated, and the skin was removed. The samples were fixed with 95% ethanol for 3 days and incubated with acetone for another 48 h. Subsequently, the samples were stained in Alcian blue solution for 3 days. Then, they were cleared with 75% ethanol three times for 1.5 h each, followed by treatment with 1.0% KOH overnight. After staining with 0.005% ARS solution for 5 h, the samples were cleared and conserved in 1.0% KOH/20% glycerol.

### Cell culture and osteogenic differentiation

BMSCs were isolated from the bone marrow of 1-month-old mouse femurs and tibias. Briefly, the femurs and tibias were dissected from soft tissue and placed in PBS containing 10% penicillin‒streptomycin. After removal of the remaining muscle and connective tissue, the epiphyses were cut away. The bones were flushed with α-MEM containing 10% penicillin‒streptomycin to remove bone marrow cells. Cells were seeded in 10 cm plates for 2 days with α-MEM containing 10% fetal bovine serum (FBS; Gibco, Grand Island, USA), the suspended cells were removed with the replacement solution, and the adherent cells were used as BMSCs. For primary osteoblasts, cells were isolated from the calvaria of neonatal mice within 3 days. Briefly, mouse calvaria was dissected aseptically and digested with 0.2% collagenase, and then, the digested cells were seeded in culture flasks with α-MEM containing 10% FBS (Gibco, Grand Island, NY, USA) and 1.0% penicillin‒streptomycin (Gibco). For osteogenic differentiation, cells were cultured in an osteogenic induction medium with α-MEM containing 10% fetal bovine serum, an additional 50 μg·mL^−1^ ascorbic acid (Sigma, A4403), and 5 mmol·L^−1^ β-glycerophosphate (Sigma, G9422).

### RNA extraction and qPCR analysis

Total RNA was extracted from osteoblasts or bone tissues with TRIzol reagent (Life Technologies, 15596018) according to the manufacturer’s instructions. RNA was reverse transcribed with the PrimeScript RT reagent Kit (TaKaRa, China) according to the manufacturer’s instructions. Quantitative reverse transcriptase-PCR (qPCR) was performed using a SYBR Premix Ex Taq II Kit (TaKaRa, China). The level of endogenous mRNA was normalized to the level of Gapdh mRNA using the 2^−ΔΔCT^ method. Specific primers are listed in Table S2.

### RNA fluorescence in situ hybridization and immunofluorescence microscopy

For specific probe synthesis, primers containing the T7 promoter were added to the HOTAIR template, and the amplification reaction was performed. The PCR products were recovered through agar gels and purified, and then, the purified PCR products were used as templates for in vitro transcription using a digoxigenin (DIG) labeling kit (Roche). RNA FISH was carried out as described in a previous study.^64^ Briefly, osteoblasts and BMSCs were rinsed gently with PBS, fixed in 4% fixative solution for 15 min at room temperature, and then permeabilized in 0.5% Triton X-100/PBS on ice for 10 min to increase the permeability of the cell membrane. Subsequently, the cells were incubated with prehybridization solution at 55 °C for 1.0 h and then incubated with a hybridization solution containing DIG-labeled HOTAIR or other RNA probes at 5–10 μg·mL^−1^ overnight at 55 °C. After hybridization, the cells were washed 3 times with prewarmed wash buffer at 55 °C for 10 min. After that, the cells were washed 3 times with buffer 1 (2× SSC and 0.01% Tween-20) at 55 °C for 10 min and then washed 3 times with buffer 2 (0.2× SSC and 0.01% Tween-20) at 55 °C for 10 min. Next, the cells were washed with 1× TBST for 5 min and incubated with 1× blocking buffer for 1 h at room temperature. Then, the cells were incubated with mouse anti-DIG antibody (1:200, Abcam, Cat. No. ab420, monoclonal) or rabbit anti-HuR antibody (1:300, Cell Signaling Technology, Cat. No. 12582) in 1× blocking buffer at room temperature for 1 h, washed 3 times with TBST for 5 min, incubated with secondary antibodies (1:300, FITC-labeled goat anti-rabbit IgG, ORIGEN, ZF-0311; 1:300, TRIC-labeled goat anti-mouse IgG, ORIGEN, ZF-0313) in 1× blocking buffer at room temperature for 1 h, and washed 3 times with 1× TBST for 5 min. Finally, cell nuclei were stained with Hoechst (Sigma-Aldrich) and washed with PBS 3 times. Cy3-labeled probes specific for 18 S RNA were used as cytoplasmic markers, and Cy3-labeled probes specific for U6 RNA were used as nuclear markers. The probe sequences for U6 and 18 S were as follows: 18 S: 5′-CTTCCTTGGATGTGGTAGCCGTTCC-3′, U6: 5′- GCAGGGGCCATGCTAATCTTCTCTGTATCG-3′. Images were taken with a Zeiss LSM 710 microscope for confocal scanning.

### Structured illumination microscopy (SIM)

SIM images of BMSCs and osteoblasts were acquired on the DeltaVision OMX V3 imaging system (Cytiva, GE Healthcare) with a ×100/1.40 NA oil objective (Olympus UPlanSApo), solid-state multimode lasers (488 nm, 405 nm, 561 nm) and electron-multiplying CCD (charge-coupled device) cameras (Evolve 512×512, Photometrics). For optimal images, immersion oils with refractive indices of 1.512 were used for BMSCs and osteoblasts on glass coverslips. The microscope is routinely calibrated with 100 nm fluorescent spheres to calculate both the lateral and axial limits of image resolution. SIM image stacks were reconstructed using WoRx 6.1.1 software (Cytiva, GE Healthcare). Conventional image stacks were processed by deconvolution methods using WoRx 6.1 software (Cytiva, GE Healthcare). The reconstructed images were further processed for maximum-intensity projections with WoRx 6.1.1 software.

### RNA immunoprecipitation

BMSCs and osteoblasts were seeded in 15 cm culture plates, harvested after reaching over 90% confluency, washed twice with ice-cold PBS and suspended in 1 mL of RNA immunoprecipitation (RIP) buffer (50 mmol·L^−1^ Tris pH 7.4, 150 mmol·L^−1^ NaCl, 0.5% Igepal, 2 mmol·L^−1^ VRC, 1 mmol·L^−1^ PMSF and 1× protease inhibitor cocktail) with RNase inhibitor (TaKaRa, 2313 A). After 30 min of incubation at 4 °C on a rotating wheel, the lysates were centrifuged at 1 000 × *g* for 10 min at 4 °C, and the supernatants were precleared with 20 μL of Protein G PLUS-agarose beads (Santa Cruz, sc-2002). The precleared supernatants were then equally divided into two parts and incubated with 30 μL of Protein G PLUS-agarose beads with rabbit anti-HuR antibody (1:300, Cell Signaling Technology, Cat. No. 12582, monoclonal) or rabbit IgG (1:500, Cell Signaling Technology, Cat. No. 3900, monoclonal) for 3 h at 4 °C, followed by washing three times with RIP buffer. Finally, TRIzol reagent was added to the precipitate, and RNA was isolated for real-time RT‒PCR. Primers used for template amplification are shown in Table S2.

### RNA pulldown

Biotinylated full-length HOTAIR was synthesized using a T7 promoter-bearing PCR product that contained full-length HOTAIR, and biotinylated miR-214 was synthesized by Ruibo Biotechnology Co., Ltd. Cell lysates from the indicated BMSCs or osteoblasts were incubated with biotin-labeled full-length HOTAIR or miR-214 (1 μmol·L^−1^) for 2 h and then with 20 μL of streptavidin-coupled agarose beads for 1 h. After extensive washing, the precipitated complexes were analyzed by real-time RT‒PCR, Western blotting and protein spectrum. The primers used for template amplification are shown in Table S2.

### MS2-based RNA immunoprecipitation (RIP) assay

The MS2-based RNA immunoprecipitation (RIP) assay was performed as described in previous studies.^65^ Osteoblasts were cotransfected with pcDNA3.1-12×MS2-Ctrl, pcDNA3.1-12×MS2-HOTAIR, and pcDNA3.1-MS2-YFP (Addgene). After 48 h, the transfected cells were collected for lysis using RIPA buffer (50 mmol·L^−1^ Tris-HCl [pH 7.4], 150 mmol·L^−1^ NaCl, 0.1% SDS, 1% NP-40, 1 mmol·L^−1^ PMSF, 1× proteinase inhibitor cocktail and 1× phosphatase inhibitor [P1260]). The lysate was incubated with rabbit anti-GFP antibody (1:200, Abcam, Cat. No. ab290, polyclonal) or rabbit IgG (1:200, Abcam, Cat. No. ab172730, monoclonal) overnight. The RNA/protein complex was recovered with protein G Dynabeads and washed with RIPA buffer several times. RNA was recovered with TRIzol and analyzed by RT‒PCR.

### Cell transfection

Plasmid and siRNA transfection was carried out with Lipofectamine 3000 (Invitrogen, L3000-015) or RNAi Max (Invitrogen, 13778-150) according to the manufacturers’ protocol. The siRNA sequences used in the study were as follows: mouse negative control (NC) siRNA: 5′-AACGUACGCGGAAUACUUCGA-3′; mouse HOTAIR: 5′-GCAGAAUUCACUCUCAAUATT-3′; and mouse *HuR*: 5′-AAGAGGCAAUUACCAGU UUCA-3′.

### Western blotting

BMSCs or primary osteoblasts were lysed in buffer (150 mmol·L^−1^ NaCl, 50 mmol·L^−1^ Tris, pH 7.8, 10% glycerol, 1 mmol·L^−1^ EGTA, 0.5% NP-40, 1 mmol·L^−1^ EDTA, 1 mmol·L^−1^ PMSF, 1x cocktail) on ice with vigorous shaking for 30 min. The lysates were subjected to Western blotting with the indicated antibody. The following antibodies were used in the study: rabbit anti-HuR antibody (1:1 000, Cell Signaling Technology, Cat. No. 12582, monoclonal), rabbit anti-Atf4 antibody (1:1 000, Cell Signaling Technology, Cat. No. 11815, monoclonal), and rabbit anti-Gapdh antibody (1:5 000, Abways, Cat. No. AB0036, monoclonal).

### ChIP assay

ChIP assays were performed using a commercial kit from Cell Signaling Technology (Cell Signaling Technology, Cat. No. 56383). Briefly, BMSCs transfected with si-HOTAIR or NC were fixed at room temperature with 1% formaldehyde for 10 min and quenched for 5 min with glycine solution. The nuclei of BMSCs were extracted, and chromatin was fragmented into lengths of 0.2-1.0 kb by ultrasound. The chromatin fragments were then immunoprecipitated with the ChIP-validated rabbit anti-H3K27me3 antibody (1:200, Cell Signaling Technology, Cat. No. 9733, monoclonal), rabbit anti-H3 antibody (1:200, Cell Signaling Technology, Cat. No. 4499, monoclonal), or rabbit IgG (1:500, Cell Signaling Technology, Cat. No. 3900, monoclonal) overnight at 4 °C, followed by incubation at 4 °C with ChIP-Grade Protein G Magnetic Beads for 2 h. After washing, the eluted DNA was purified and assessed by qRT‒PCR to measure the amount of enrichment of the region of the *Runx2* or *Sp7* gene promoter. Histone H3 antibody pulldown for enrichment served as a positive control. The primers used for qRT‒PCR are shown in Table S2.

### Statistical analysis

For statistical analysis, all quantitative data are presented as the mean ± SEM. Student’s t test was used for statistical evaluations of two group comparisons. Statistical differences among groups were analyzed by 1-way analysis of variance (ANOVA) or 2-way ANOVA (if there were 2 factor levels), followed by a post hoc test to determine group differences in the study parameters. All statistical analyses were performed with Prism software (GraphPad Prism for Windows, version 8.0, Nashville, USA). Differences were considered significant at ^*^P < 0.05, ^**^P < 0.01, and ^***^P < 0.001.

### Supplementary information

Supplementary Table 2

Supplementary Figures

Supplementary Table 1
