# Supplementary material for: HuR-mediated nucleocytoplasmic translocation of HOTAIR relieves its inhibition of osteogenic differentiation and promotes bone formation
Source: Bone Res. 2023 Oct 23;11:53. doi: 10.1038/s41413-023-00289-2 (PMC10593784; doi:10.1038/s41413-023-00289-2)
Supplement: Supplementary file 1 — Supplementary Table 2 [file 41413_2023_289_MOESM1_ESM.docx]

| **The sequence of primers were used for detecting mRNA expression by quantitative PCR** | |
| --- | --- |
| Mmu-*Gapdh*-F | TCACCACCATGGAGAAGGC |
| Mmu-*Gapdh*-R | GCTAAGCAGTTGGTGGTGCA |
| Mmu-*Bglap*-F | CTGACCTCACAGATCCCAAGC |
| Mmu-*Bglap*-R | TGGTCTGATAGCTCGTCACAAG |
| Mmu-*Alp*-F | ATCTTTGGTCTGGCTCCCATG |
| Mmu-*Alp*-R | TTTCCCGTTCACCGTCCAC |
| Mmu-*Col1a1*-F | GGGACCAGGAGGACCAGGAAGT |
| Mmu-*Col1a1*-R | GGAGGGCGAGTGCTGTGCTTT |
| Mmu-HOTAIR-F | GGTCCCCAACATCGGTAGAA |
| Mmu-HOTAIR-R | ACTGGGGTTTGTCTGGAGTT |
| Mmu-*Runx2*-F | CCAGAATGATGGTGTTGACG |
| Mmu-*Runx2*-R | GGTTGCAAGATCATGACTAGGG |
| Mmu-*Sp7*-F | TGAGGAAGAAGCCCATTCAC |
| Mmu-*Sp7*-R | ACTTCTTCTCCCGGGTGTG |
| Mmu-miR-214-F | GACAGCAGGCACAGACA |
| Mmu-miR-214-R | GTGCAGGGTCCGAGGT |
| Mmu-miR-214-RT | GTCGTATCCAGTGCAGGGTCCGAGGTA  TTCGCACTGGATACGACACTGCC |
| **Primer sequences were used to detect DNA fragments enriched by CHIP PCR** | |
| *Runx2*-2kb-F | GTACAAGGAATCGCAGCACT |
| *Runx2*-2kb-R | GACATAAAAGACCTGGGCACTCT |
| *Runx2*-1kb-F | AGGACTTTGGGTGCAGTTCT |
| *Runx2*-1kb-R | CCCCCTCTATTCTGAGCTATGGA |
| *Runx2-*TSS-F | TCAACTGAGTGTGTGGCGTT |
| *Runx2*-TSS-R | GAAATCCGAAGCAGCCTTGC |
| *Sp7*-2kb-F | GTGAGCCCAACATTCAAGGAA |
| *Sp7*-2kb-R | CTGGGCGTCCTCTTTTCTGTC |
| *Sp7*-1kb-F | GGATGATGTGCCAAGTGTGC |
| *Sp7*-1kb-R | CCACAGCAAGCTTTTCCCAC |
| *Sp7*-TSS-F | ATGATCACACAATGGGGGCA |
| *Sp7*-TSSr-R | ACCCTGAACTAGTGGTGGCTA |

**Supplementary Table 2. mRNA and CHIP PCR primers are used as follows**
