# Supplementary material for: HuR-mediated nucleocytoplasmic translocation of HOTAIR relieves its inhibition of osteogenic differentiation and promotes bone formation
Source: Bone Res. 2023 Oct 23;11:53. doi: 10.1038/s41413-023-00289-2 (PMC10593784; doi:10.1038/s41413-023-00289-2)
Supplement: Supplementary file 2 — Supplementary Figures [file 41413_2023_289_MOESM2_ESM.pptx]

## Slide 1
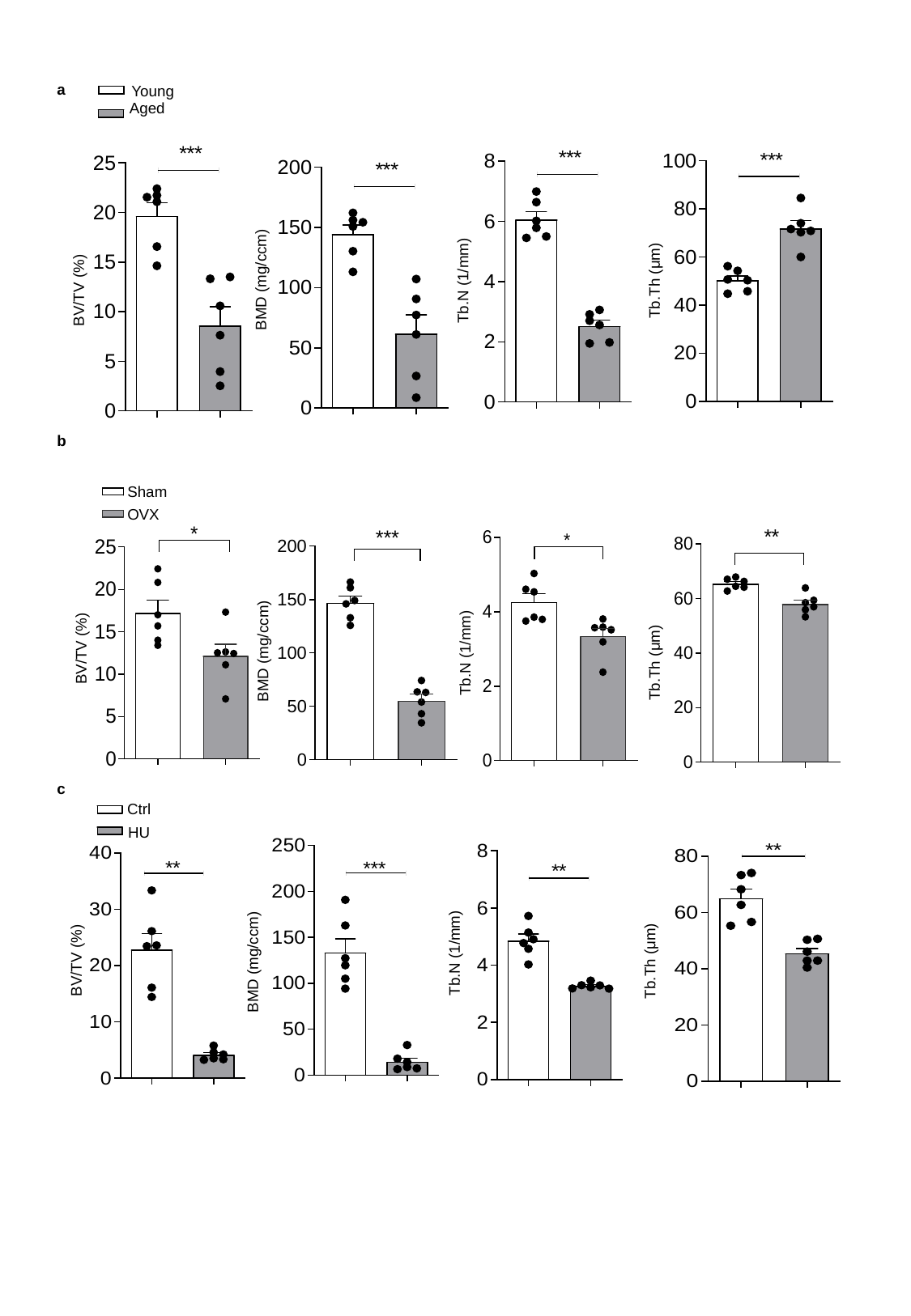

BV/TV (%)
Young
Aged
a
Tb.Th (μm)
Tb.N (1/mm)
BMD (mg/ccm)
b
BV/TV (%)
Sham
OVX
Tb.N (1/mm)
Tb.Th (μm)
BMD (mg/ccm)
c
BV/TV (%)
Ctrl
HU
BMD (mg/ccm)
Tb.N (1/mm)
Tb.Th (μm)

## Slide 2
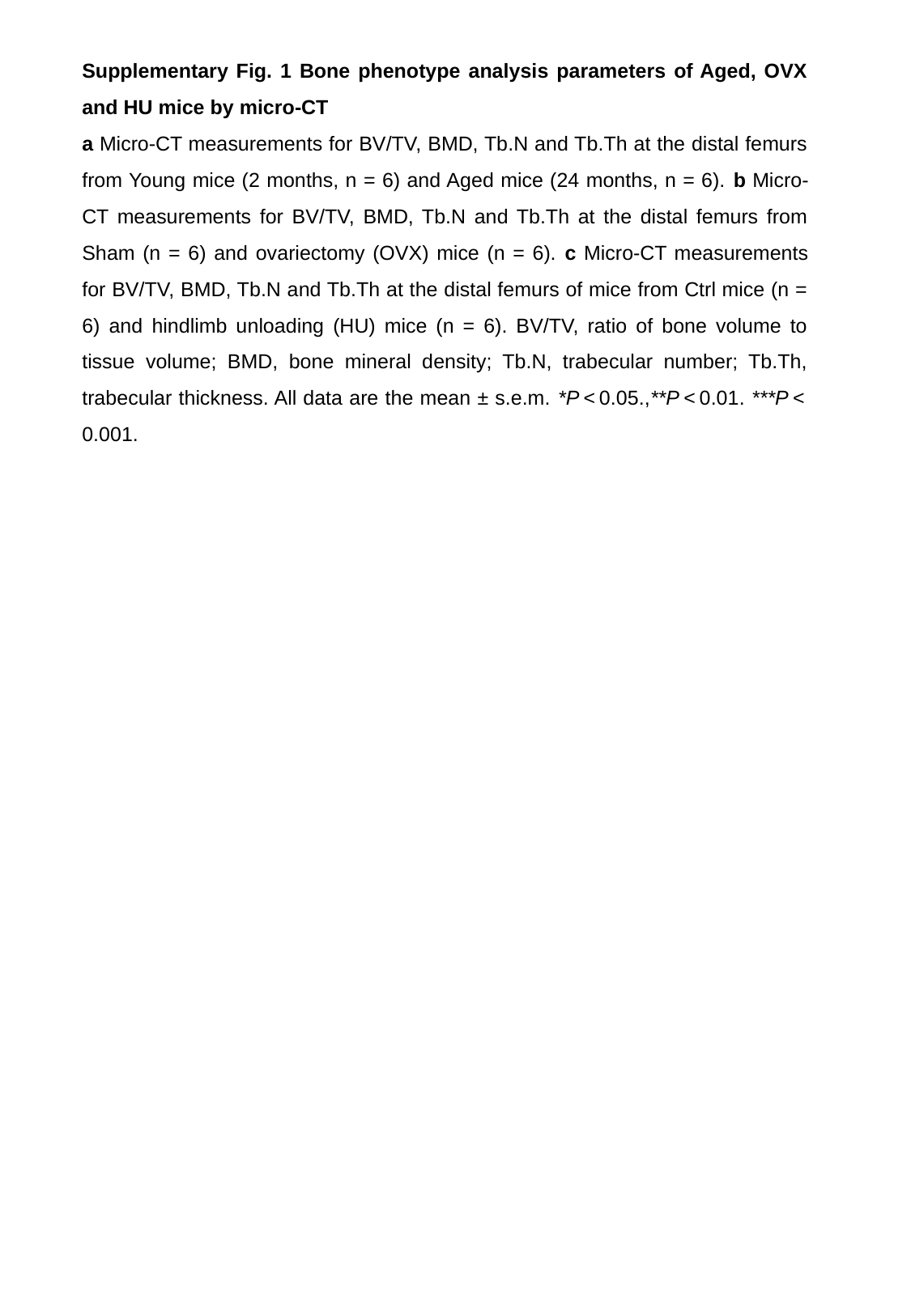

Supplementary Fig. 1 Bone phenotype analysis parameters of Aged, OVX and HU mice by micro-CT
a Micro-CT measurements for BV/TV, BMD, Tb.N and Tb.Th at the distal femurs from Young mice (2 months, n = 6) and Aged mice (24 months, n = 6). b Micro-CT measurements for BV/TV, BMD, Tb.N and Tb.Th at the distal femurs from Sham (n = 6) and ovariectomy (OVX) mice (n = 6). c Micro-CT measurements for BV/TV, BMD, Tb.N and Tb.Th at the distal femurs of mice from Ctrl mice (n = 6) and hindlimb unloading (HU) mice (n = 6). BV/TV, ratio of bone volume to tissue volume; BMD, bone mineral density; Tb.N, trabecular number; Tb.Th, trabecular thickness. All data are the mean ± s.e.m. *P < 0.05.,**P < 0.01. ***P < 0.001.

## Slide 3
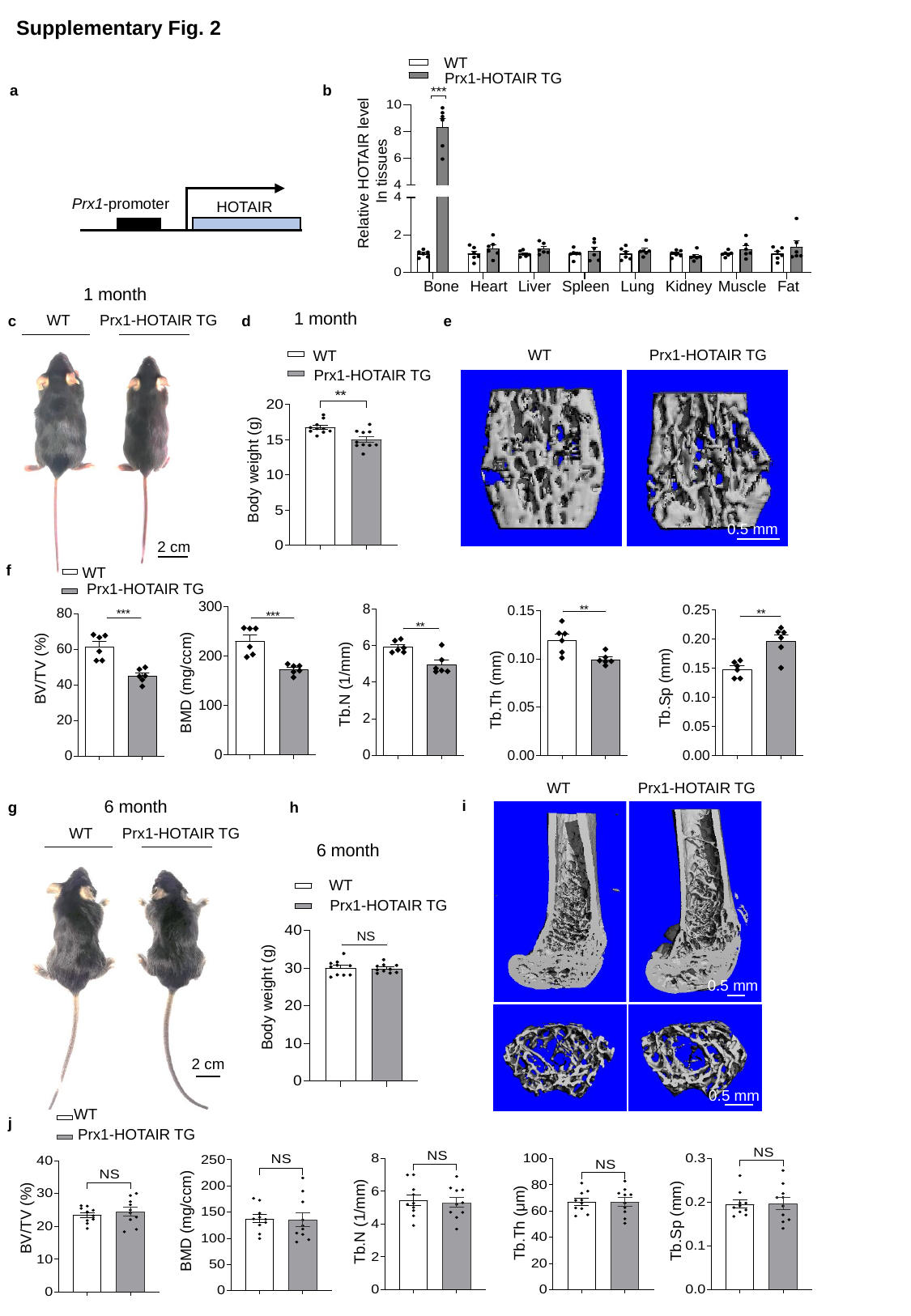

Supplementary Fig. 2
WT
Prx1-HOTAIR TG
Relative HOTAIR level
In tissues
Bone
Heart
Liver
Spleen
Lung
Kidney
Muscle
Fat
a
b
Prx1-promoter
HOTAIR
1 month
2 cm
WT Prx1-HOTAIR TG
1 month
WT
Prx1-HOTAIR TG
Body weight (g)
c
d
e
WT
Prx1-HOTAIR TG
0.5 mm
f
WT
Prx1-HOTAIR TG
BV/TV (%)
BMD (mg/ccm)
Tb.N (1/mm)
Tb.Sp (mm)
Tb.Th (mm)
WT
Prx1-HOTAIR TG
0.5 mm
0.5 mm
6 month
WT Prx1-HOTAIR TG
2 cm
i
g
h
6 month
WT
Prx1-HOTAIR TG
Body weight (g)
WT
BV/TV (%)
j
Prx1-HOTAIR TG
Tb.Sp (mm)
Tb.Th (μm)
Tb.N (1/mm)
BMD (mg/ccm)

## Slide 4
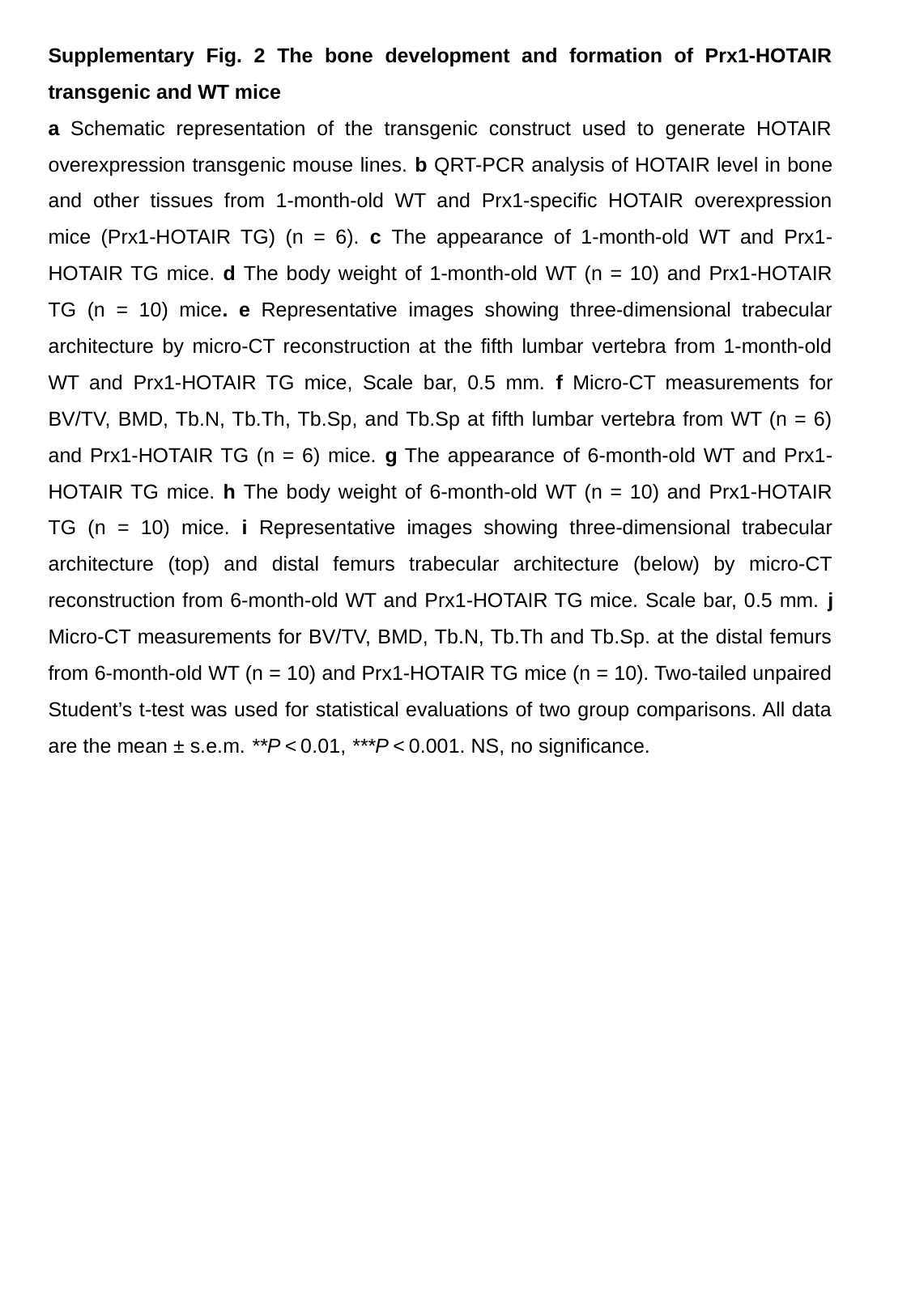

Supplementary Fig. 2 The bone development and formation of Prx1-HOTAIR transgenic and WT mice
a Schematic representation of the transgenic construct used to generate HOTAIR overexpression transgenic mouse lines. b QRT-PCR analysis of HOTAIR level in bone and other tissues from 1-month-old WT and Prx1-speciﬁc HOTAIR overexpression mice (Prx1-HOTAIR TG) (n = 6). c The appearance of 1-month-old WT and Prx1-HOTAIR TG mice. d The body weight of 1-month-old WT (n = 10) and Prx1-HOTAIR TG (n = 10) mice. e Representative images showing three-dimensional trabecular architecture by micro-CT reconstruction at the fifth lumbar vertebra from 1-month-old WT and Prx1-HOTAIR TG mice, Scale bar, 0.5 mm. f Micro-CT measurements for BV/TV, BMD, Tb.N, Tb.Th, Tb.Sp, and Tb.Sp at fifth lumbar vertebra from WT (n = 6) and Prx1-HOTAIR TG (n = 6) mice. g The appearance of 6-month-old WT and Prx1-HOTAIR TG mice. h The body weight of 6-month-old WT (n = 10) and Prx1-HOTAIR TG (n = 10) mice. i Representative images showing three-dimensional trabecular architecture (top) and distal femurs trabecular architecture (below) by micro-CT reconstruction from 6-month-old WT and Prx1-HOTAIR TG mice. Scale bar, 0.5 mm. j Micro-CT measurements for BV/TV, BMD, Tb.N, Tb.Th and Tb.Sp. at the distal femurs from 6-month-old WT (n = 10) and Prx1-HOTAIR TG mice (n = 10). Two-tailed unpaired Student’s t-test was used for statistical evaluations of two group comparisons. All data are the mean ± s.e.m. **P < 0.01, ***P < 0.001. NS, no significance.

## Slide 5
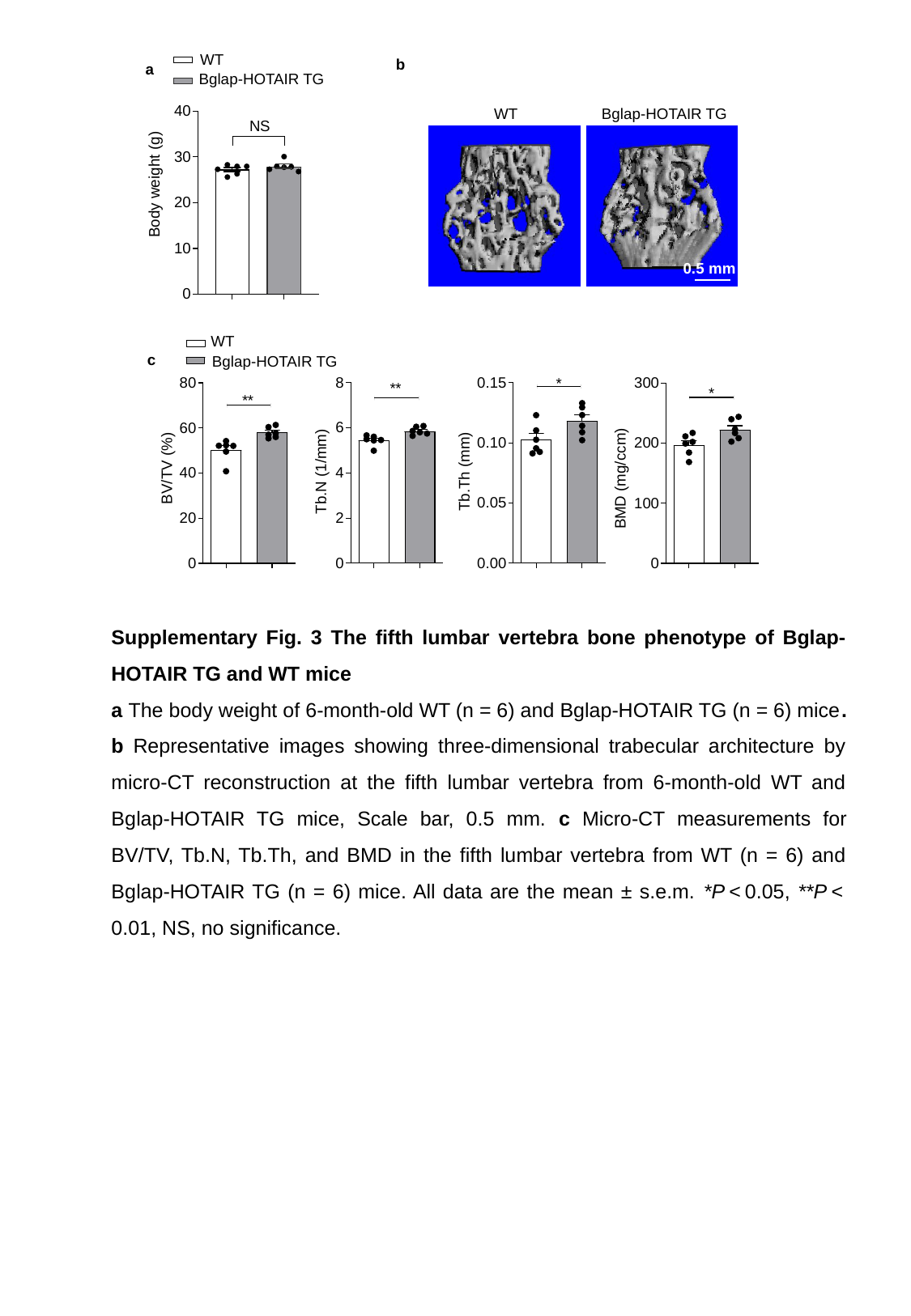

WT
Bglap-HOTAIR TG
Body weight (g)
b
a
WT
Bglap-HOTAIR TG
0.5 mm
WT
Bglap-HOTAIR TG
BV/TV (%)
c
Tb.N (1/mm)
Tb.Th (mm)
BMD (mg/ccm)
Supplementary Fig. 3 The fifth lumbar vertebra bone phenotype of Bglap-HOTAIR TG and WT mice
a The body weight of 6-month-old WT (n = 6) and Bglap-HOTAIR TG (n = 6) mice. b Representative images showing three-dimensional trabecular architecture by micro-CT reconstruction at the fifth lumbar vertebra from 6-month-old WT and Bglap-HOTAIR TG mice, Scale bar, 0.5 mm. c Micro-CT measurements for BV/TV, Tb.N, Tb.Th, and BMD in the fifth lumbar vertebra from WT (n = 6) and Bglap-HOTAIR TG (n = 6) mice. All data are the mean ± s.e.m. *P < 0.05, **P < 0.01, NS, no significance.

## Slide 6
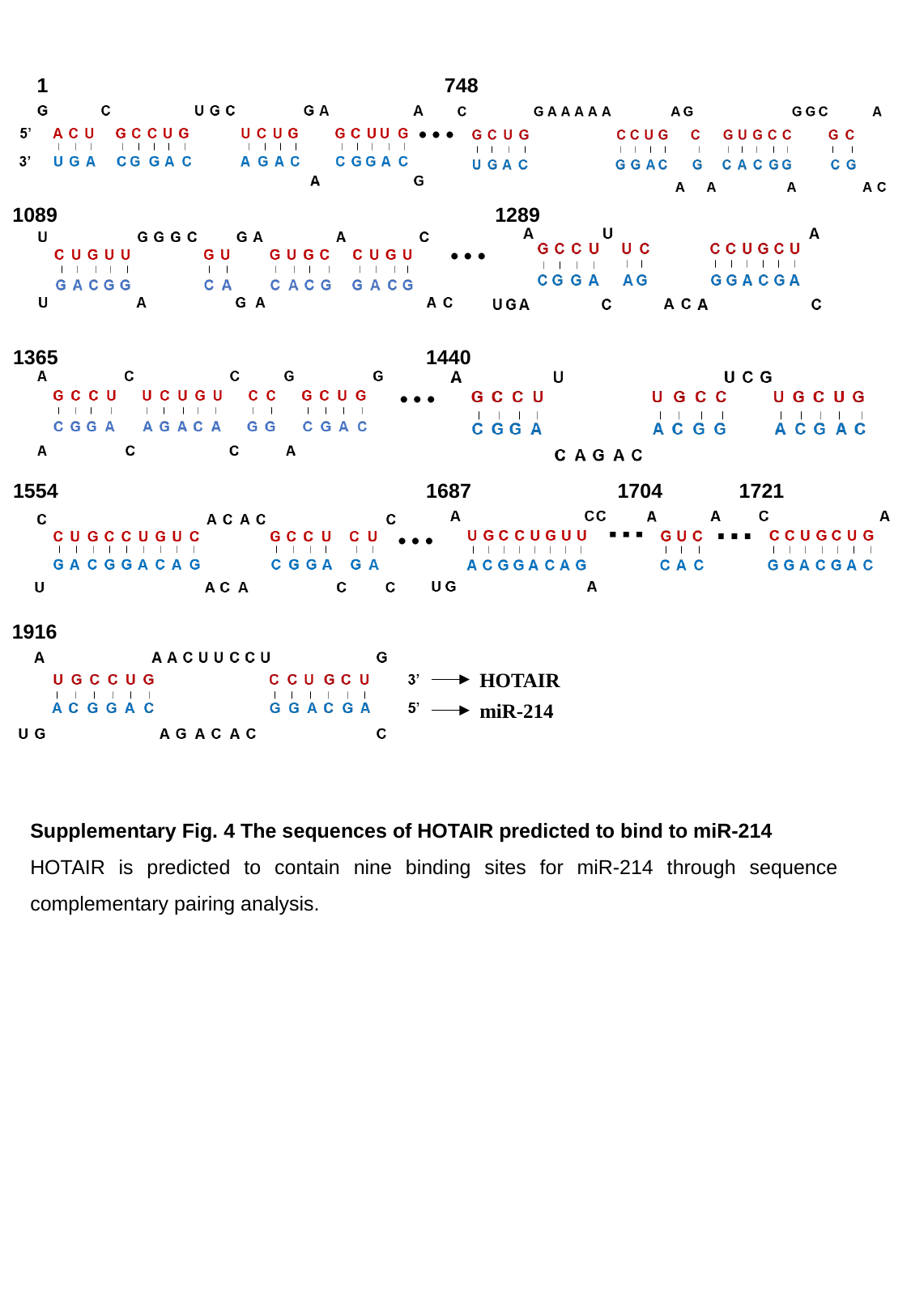

1
748
…
1089
1289
…
1365
1440
…
1554
1687
1704
1721
…
1916
HOTAIR
miR-214
Supplementary Fig. 4 The sequences of HOTAIR predicted to bind to miR-214
HOTAIR is predicted to contain nine binding sites for miR-214 through sequence complementary pairing analysis.

## Slide 7
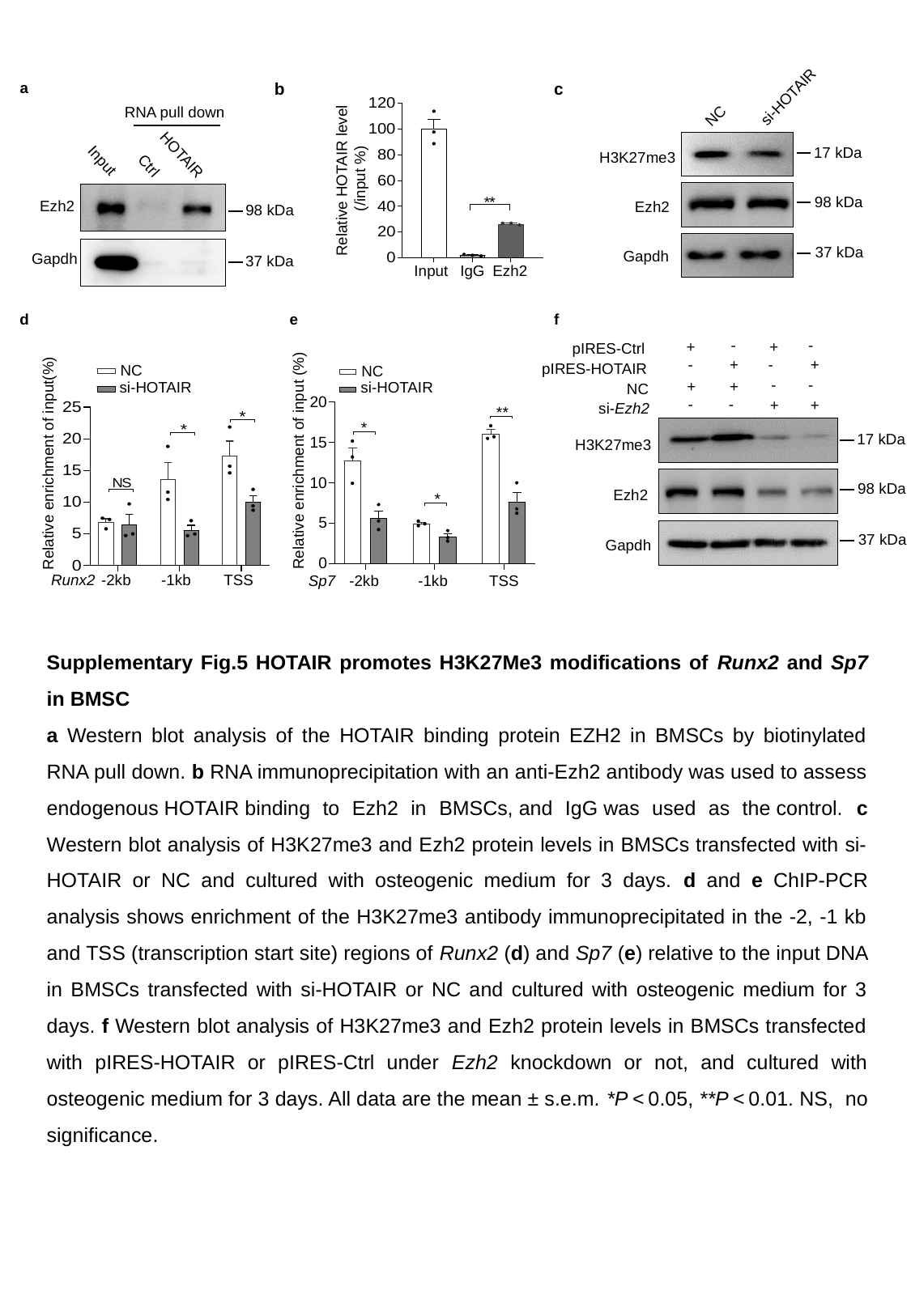

a
b
c
si-HOTAIR
Relative HOTAIR level
(/input %)
Input
IgG
Ezh2
NC
H3K27me3
Ezh2
Gapdh
RNA pull down
HOTAIR
Input
Ctrl
Ezh2
98 kDa
Gapdh
37 kDa
17 kDa
98 kDa
37 kDa
d
e
f
Relative enrichment of input (%)
Sp7
-2kb
-1kb
TSS
NC
si-HOTAIR
-
-
+
+
pIRES-Ctrl
-
+
-
+
pIRES-HOTAIR
-
-
+
+
NC
-
-
+
+
si-Ezh2
H3K27me3
Ezh2
Gapdh
NC
si-HOTAIR
Relative enrichment of input(%)
Runx2
-2kb
-1kb
TSS
17 kDa
98 kDa
37 kDa
Supplementary Fig.5 HOTAIR promotes H3K27Me3 modifications of Runx2 and Sp7 in BMSC
a Western blot analysis of the HOTAIR binding protein EZH2 in BMSCs by biotinylated RNA pull down. b RNA immunoprecipitation with an anti-Ezh2 antibody was used to assess endogenous HOTAIR binding to Ezh2 in BMSCs, and IgG was used as the control. c Western blot analysis of H3K27me3 and Ezh2 protein levels in BMSCs transfected with si-HOTAIR or NC and cultured with osteogenic medium for 3 days. d and e ChIP-PCR analysis shows enrichment of the H3K27me3 antibody immunoprecipitated in the -2, -1 kb and TSS (transcription start site) regions of Runx2 (d) and Sp7 (e) relative to the input DNA in BMSCs transfected with si-HOTAIR or NC and cultured with osteogenic medium for 3 days. f Western blot analysis of H3K27me3 and Ezh2 protein levels in BMSCs transfected with pIRES-HOTAIR or pIRES-Ctrl under Ezh2 knockdown or not, and cultured with osteogenic medium for 3 days. All data are the mean ± s.e.m. *P < 0.05, **P < 0.01. NS, no significance.

## Slide 8
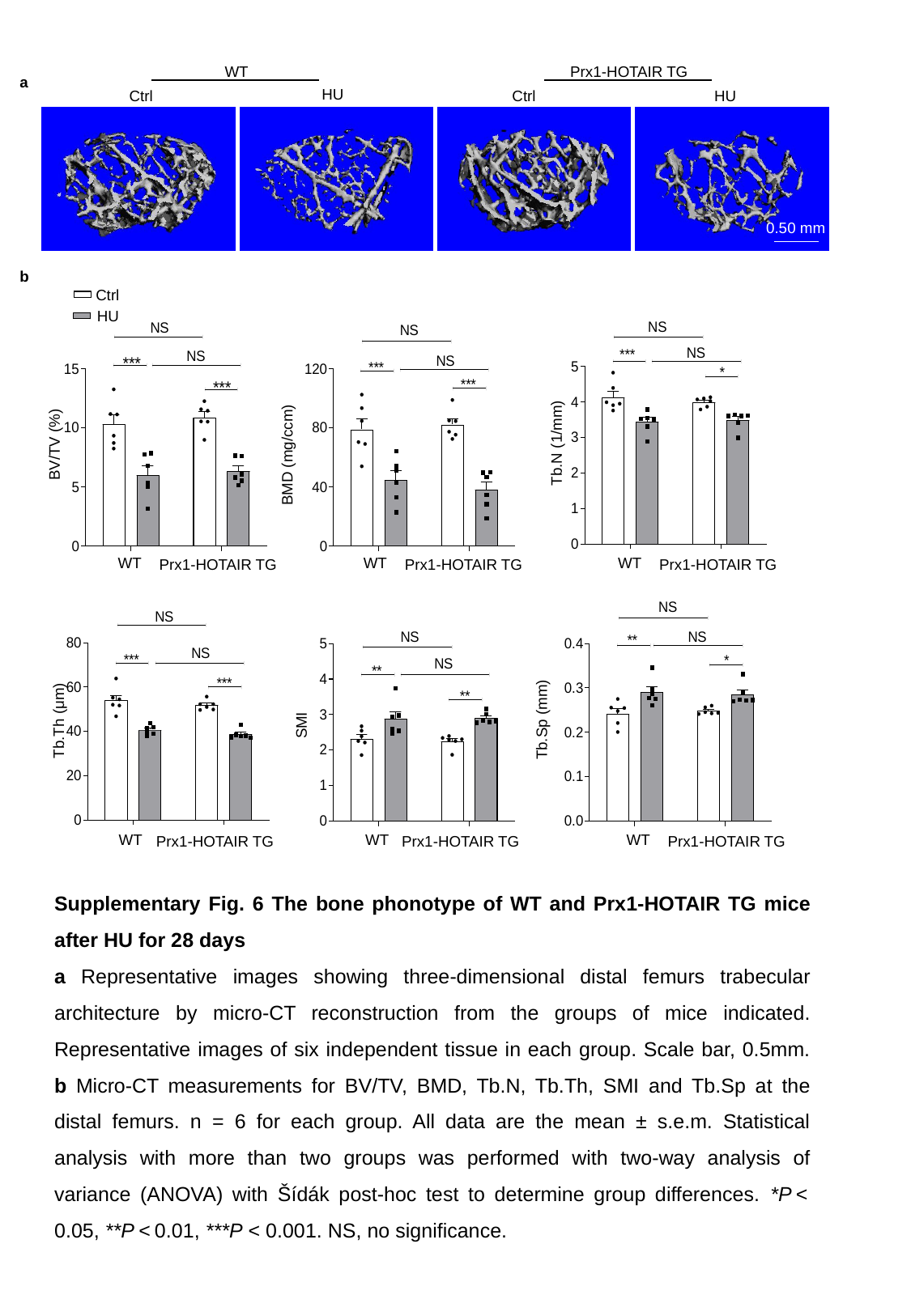

WT
Prx1-HOTAIR TG
HU
Ctrl
HU
Ctrl
a
0.50 mm
b
Ctrl
HU
BV/TV (%)
Tb.N (1/mm)
BMD (mg/ccm)
WT
WT
WT
Prx1-HOTAIR TG
Prx1-HOTAIR TG
Prx1-HOTAIR TG
Tb.Sp (mm)
Tb.Th (μm)
SMI
WT
WT
WT
Prx1-HOTAIR TG
Prx1-HOTAIR TG
Prx1-HOTAIR TG
Supplementary Fig. 6 The bone phonotype of WT and Prx1-HOTAIR TG mice after HU for 28 days
a Representative images showing three-dimensional distal femurs trabecular architecture by micro-CT reconstruction from the groups of mice indicated. Representative images of six independent tissue in each group. Scale bar, 0.5mm. b Micro-CT measurements for BV/TV, BMD, Tb.N, Tb.Th, SMI and Tb.Sp at the distal femurs. n = 6 for each group. All data are the mean ± s.e.m. Statistical analysis with more than two groups was performed with two-way analysis of variance (ANOVA) with Šídák post-hoc test to determine group differences. *P < 0.05, **P < 0.01, ***P < 0.001. NS, no significance.
