# Supplementary material for: HuR-mediated nucleocytoplasmic translocation of HOTAIR relieves its inhibition of osteogenic differentiation and promotes bone formation
Source: Bone Res. 2023 Oct 23;11:53. doi: 10.1038/s41413-023-00289-2 (PMC10593784; doi:10.1038/s41413-023-00289-2)
Supplement: Supplementary file 3 — Supplementary Table 1 [file 41413_2023_289_MOESM3_ESM.pdf]

Supplementary Table 1. Mass spectrometry analysis of HOTAIR interaction proteins

Accession	Description	Coverage	# Peptides	# PSMs	# Unique Peptides	# AAs	MW [kDa]	calc. pI	Area: F3: Sample	Score Mascot
4885049	actin, alpha cardiac muscle 1 proprotein	53.0504	18	52	7	377	42	5.39	317361168	667
4885377	histone H1.3	37.10407	13	25	1	221	22.3	11.02	57168791	528
281604138	serine/threonine-protein phosphatase PGAM5, mitochondrial isoform 2	35.41667	7	10	1	288	31.9	8.68	12229075	148
154937327	zinc finger protein 593	33.58209	3	3	3	134	15.2	9.82	5926639.6	35
119943100	precursor	33.39518	11	16	11	539	58.2	7.64	45505304	323
10800140	histone H2B type 1-B	30.15873	3	7	1	126	13.9	10.32	8579174.2	261
14917109	AP-2 complex subunit mu isoform a	29.88506	11	14	1	435	49.6	9.54	10173366	137
148747209	nucleolar protein 16 isoform 3	26.96629	3	4	3	178	21.2	9.94	16721116	98
4885373	histone H1.1	26.51163	7	12	1	215	21.8	10.99	4432695.4	300
26051231	serine beta-lactamase-like protein LACTB, mitochondrial isoform a	20.84095	9	10	9	547	60.7	8.53	35488284	183
109948279	galectin-7	20.58824	2	3	2	136	15.1	7.62	2622715	37
14043072	heterogeneous nuclear ribonucleoproteins A2/B1 isoform B1	20.3966	3	5	3	353	37.4	8.95	9457438.3	25
7669477	double-stranded RNA-specific editase 1 isoform 2	19.97301	8	9	8	741	80.7	9.01	18426712	69
4506643	60S ribosomal protein L37a	19.56522	1	2	1	92	10.3	10.43	28268149	37
4506753	ruvB-like 1	19.51754	6	6	6	456	50.2	6.42	15946394	99
4505815	phosphatidylinositol 4-phosphate 5-kinase type-1 alpha isoform 2	19.48998	7	9	6	549	61.1	8.34	37219903	210
14149704	oxysterol-binding protein-related protein 3 isoform a	19.27847	12	12	12	887	101.2	6.87	19045072	123
5803121	protein disulfide-isomerase A5 precursor	18.68979	7	8	7	519	59.6	7.91	23160451	97
15431306	60S ribosomal protein L8	18.28794	4	6	4	257	28	11.03	6475804.6	85
218563737	chromatin complexes subunit BAP18 isoform 1	18.22917	2	2	2	192	19.9	5.66	5284633.7	0
4505895	pleiotropic regulator 1 isoform 1	17.89883	4	4	4	514	57.2	9.17	8218430.1	51
372466572	keratin, type II cytoskeletal 8 isoform 1	17.80822	11	28	5	511	56.6	5.43	10020385	472
13129104	coiled-coil domain-containing protein 86	17.77778	4	4	4	360	40.2	10.33	16018298	99
33356552	G patch domain-containing protein 4 isoform 1	17.6	5	5	5	375	42.6	9.31	9617592.1	39
224809478	ankycorbin isoform d	17.39573	11	12	11	983	110.4	6.11	12027137	89
392494084	eukaryotic translation initiation factor 6 isoform a	17.14286	2	3	2	245	26.6	4.68	16977857	44
14043070	heterogeneous nuclear ribonucleoprotein A1 isoform b	16.93548	4	6	4	372	38.7	9.13	49014769	117
10835240	high mobility group nucleosome-binding domain-containing protein 4	16.66667	1	2	1	90	9.5	10.48	17462119	30
71772583	40S ribosomal protein S29 isoform 2	16.41791	1	1	1	67	8.1	10.02	3426438.2	0
18104948	60S ribosomal protein L21	16.25	2	3	2	160	18.6	10.49	16531967	43
239787829	glioma tumor suppressor candidate region gene 2 protein	15.69038	4	4	4	478	54.4	10.32	10365563	48
156415992	dimethyladenosine transferase 1, mitochondrial	15.60694	3	3	3	346	39.5	9.26	10674962	81
38201714	ELAV-like protein 1	15.33742	3	5	3	326	36.1	9.17	8711663.9	43
13129018	gamma-glutamylcyclotransferase isoform 1	14.89362	2	2	2	188	21	5.14	2785233.7	0
74136883	heterogeneous nuclear ribonucleoprotein U isoform a	14.42424	8	11	8	825	90.5	6	70382487	169
4758862	eukaryotic translation elongation factor 1 epsilon-1 isoform 1	14.36782	1	1	1	174	19.8	8.54	5528710.2	0
15149476	arginine--tRNA ligase, cytoplasmic	14.09091	7	7	7	660	75.3	6.68	15276221	73

5730023	ruvB-like 2	14.03888	5	6	5	463	51.1	5.64	13900629	55
323276700	40S ribosomal protein S10	13.93939	2	2	2	165	18.9	10.15	7364529.5	58
160707950	methyl-CpG-binding protein 2 isoform 2	13.65462	4	5	4	498	53.3	9.88	6668239.6	38
4505467	5'-nucleotidase isoform 1 preproprotein	13.58885	4	5	4	574	63.3	7.03	14566236	116
38679888	ras-related protein Rab-6A isoform b	13.46154	1	1	1	208	23.6	5.54	14808249	0
4502303	ATP synthase subunit O, mitochondrial precursor	13.14554	2	2	2	213	23.3	9.96	5836277.1	40
4507217	signal recognition particle 9 kDa protein isoform 2	12.7907	1	1	1	86	10.1	7.97	21443762	47
7661592	histone chaperone ASF1A	12.7451	1	1	1	204	23	4.41	9277162.7	0
13236553	nucleolar protein 12	12.67606	1	2	1	213	24.6	10.23	9404138.6	20
31083351	exocyst complex component 3	12.61745	7	7	7	745	85.5	6.29	8815418.9	66
228008291	heterogeneous nuclear ribonucleoprotein Q isoform 1	12.35955	5	5	4	623	69.6	8.59	17832756	101
4506607	60S ribosomal protein L18 isoform 1	12.23404	2	2	2	188	21.6	11.72	8272356	64
4506145	trypsin-1 preproprotein	12.14575	2	3	2	247	26.5	6.51	27082789	27
156151394	heterogeneous nuclear ribonucleoprotein R isoform 1	12.10692	5	5	4	636	71.2	8.13	13045433	80
100913206	ATP-dependent RNA helicase A	11.88976	10	11	10	1270	140.9	6.84	20834536	195
4759098	transformer-2 protein homolog beta isoform 1	11.80556	3	3	3	288	33.6	11.25	11406033	70
42491362	inhibitor of nuclear factor kappa-B kinase-interacting protein isoform 2	11.71429	4	5	2	350	39.3	9.17	7670000.9	150
5454106	transcription initiation factor TFIID subunit 10	11.46789	1	1	1	218	21.7	6.57	2856634.1	98
50980307	myosin phosphatase Rho-interacting protein isoform 1	11.46435	7	8	7	1038	118	6.39	9275290.2	143
131412225	keratin, type I cytoskeletal 13 isoform a	11.35371	7	12	1	458	49.5	4.96	1701781.1	211
109715866	FERM domain-containing protein 6 isoform 1	11.23779	5	6	5	614	70.9	7.46	8028515.8	120
14141152	heterogeneous nuclear ribonucleoprotein M isoform a	11.23288	6	6	6	730	77.5	8.7	21472614	82
189083778	gelsolin isoform b	11.21751	5	6	5	731	80.6	5.85	10808641	74
4757718	actin-like protein 6A isoform 1	11.18881	2	3	2	429	47.4	5.6	25124002	17
7657198	probable dimethyladenosine transferase	11.18211	3	3	3	313	35.2	9.99	4131362.4	38
31563534	replication factor C subunit 2 isoform 1	11.01695	3	3	3	354	39.1	6.44	6637324.3	34
56699409	RNA-binding motif protein, X chromosome isoform 1	10.99744	3	4	3	391	42.3	10.05	13569689	111
6912420	heparan sulfate 2-O-sulfotransferase 1 isoform 1	10.95506	3	3	3	356	41.9	8.69	10057403	48
58218968	calmodulin	10.73826	1	1	1	149	16.8	4.22	17174379	42
40354195	keratin, type I cytoskeletal 18	10.69767	4	4	4	430	48	5.45	19828324	42
345441819	kinesin-like protein KIF2A isoform 4	10.48035	5	5	5	687	78	6.68	6529826.3	47
153252081	ribonuclease 7 precursor	10.25641	2	2	2	156	17.4	9.66	2785946.6	27
82659087	double-stranded RNA-binding protein Staufen homolog 1 isoform b	10.2253	3	3	3	577	63.1	9.44	4247964.3	30
4505581	interferon-inducible double stranded RNA-dependent protein kinase activator A isoform 1	10.22364	2	2	2	313	34.4	8.41	8194327.3	65
311078508	histone-binding protein RBBP7 isoform 1	10.02132	3	3	2	469	52.3	5.27	2896706.7	23
16933546	60S acidic ribosomal protein P0	9.77918	1	1	1	317	34.3	5.97	6757242.4	19
116812591	protein RER1	9.693878	1	1	1	196	22.9	9.54	2609375.4	0
45593130	guanine nucleotide-binding protein-like 3 isoform 1	9.289617	3	3	3	549	62	9.16	9495106.3	32
8923900	N-acylneuraminate cytidylyltransferase	9.21659	2	2	2	434	48.3	7.93	6762962.4	24

4885245	fos-related antigen 2	9.202454	1	1	1	326	35.2	7.49	3703982.8	35
94721241	isoleucine--tRNA ligase, cytoplasmic	9.11252	9	9	9	1262	144.4	6.15	10006288	54
104485446	transcription initiation factor TFIID subunit 8	9.032258	2	2	2	310	34.2	6.46	3586604.4	39
68303561	proteasome subunit alpha type-7-like isoform 1	8.984375	1	1	1	256	28.5	8.98	1320098	0
21361348	U3 small nucleolar RNA-associated protein 14 homolog A isoform 1	8.949416	4	4	4	771	87.9	7.87	7987592.7	106
31881687	replication factor C subunit 4	8.815427	2	2	2	363	39.7	8.02	8784895.8	37
16905517	serine/arginine-rich splicing factor 10 isoform 2	8.778626	2	2	2	262	31.3	11.27	6080656.1	34
16950591	28S ribosomal protein S12, mitochondrial precursor	8.695652	2	2	2	138	15.2	10.29	1900807.7	0
23110935	proteasome subunit alpha type-1 isoform 1	8.550186	2	2	2	269	30.2	6.99	2547614.7	25
11641247	Golgi-associated plant pathogenesis-related protein 1	8.441558	1	1	1	154	17.2	9.41		49
19923732	general transcription factor IIH subunit 3 isoform a	8.441558	2	2	2	308	34.4	7.02	3817445.2	45
7706351	peptidyl-tRNA hydrolase 2, mitochondrial precursor	8.379888	1	1	1	179	19.2	8.73		0
154448890	THO complex subunit 1	8.371385	3	4	3	657	75.6	4.98	10031543	62
22538467	proteasome subunit beta type-4	8.333333	1	1	1	264	29.2	5.97	4667960.3	0
365192532	myosin-10 isoform 1	8.1714	15	27	6	2007	232.4	5.55	4106588.4	387
190014588	PC4 and SFRS1-interacting protein isoform 2	8.113208	3	3	3	530	60.1	9.13	6195644.1	33
23503295	casein kinase II subunit beta	7.906977	1	2	1	215	24.9	5.55	10532418	25
27545326	SWI/SNF-related matrix-associated actin-dependent regulator of chromatin subfamily B member 1 isoform a	7.792208	2	2	2	385	44.1	6.23	6495619.3	29
4504809	transcription factor jun-B	7.78098	2	2	2	347	35.9	9.22	7421551.1	36
67189747	60S ribosomal protein L6	7.638889	2	2	2	288	32.7	10.58	8323202.3	0
323668286	TBC1 domain family member 10A isoform 1	7.572816	2	2	2	515	57.9	8.5	8703003.9	40
6005884	translocon-associated protein subunit gamma	7.567568	1	1	1	185	21.1	9.61		0
38327634	ATP-dependent RNA helicase DDX18	7.462687	4	4	4	670	75.4	9.5	4977529.5	64
29837655	polymerase delta-interacting protein 3 isoform 1	7.36342	2	2	2	421	46.1	9.99	2421119.4	29
4506489	replication factor C subunit 3 isoform 1	7.303371	2	2	2	356	40.5	8.34	2492517.2	32
52627149	telomeric repeat-binding factor 2-interacting protein 1	7.26817	1	1	1	399	44.2	4.73	3949276.5	28
4826960	glutamine--tRNA ligase isoform a	7.225806	4	4	4	775	87.7	7.15	6282861.8	0
28558975	mediator of RNA polymerase II transcription subunit 17	7.219662	3	3	3	651	72.8	7.44	4369246.5	30
62865614	adenylate kinase isoenzyme 6 isoform a	7.19697	1	1	1	264	29	8.66	11818687	0
117306176	kallikrein-5 preproprotein	7.167235	1	1	1	293	32	8.27	5105807.4	0
4507513	metalloproteinase inhibitor 3 precursor	7.109005	2	2	2	211	24.1	8.72	23296088	38
4506387	UV excision repair protein RAD23 homolog B isoform 1	7.090465	2	2	2	409	43.1	4.84	4124901.2	35
118344456	U3 small nucleolar RNA-associated protein 18 homolog	7.014388	3	3	3	556	62	8.76	3744263.2	63
4504425	high mobility group protein B1	6.976744	1	1	1	215	24.9	5.74	9645036.4	0
19115964	1-phosphatidylinositol 4,5-bisphosphate phosphodiesterase delta-3	6.970849	4	4	4	789	89.2	6.98	3410426.5	32
193211480	superkiller viralicidic activity 2-like 2	6.909789	6	6	6	1042	117.7	6.52	17557584	95
30410788	tumor suppressor candidate 3 isoform a precursor	6.896552	2	2	2	348	39.7	9.91	9597306.1	37
4506695	40S ribosomal protein S19	6.896552	1	1	1	145	16.1	10.32	8068652	44
4826742	glutamine--fructose-6-phosphate aminotransferase [isomerizing] 2	6.891496	3	3	2	682	76.9	7.37	3002651	44

4885105	chromatin assembly factor 1 subunit B	6.618962	3	3	3	559	61.5	7.5	4455489.8	49
223718046	exocyst complex component 7 isoform 4	6.530612	3	3	3	735	83.3	6.79	3685188.1	49
13236587	transmembrane protein 43	6.5	1	1	1	400	44.8	8.13	6624484.2	34
4506787	ras GTPase-activating-like protein IQGAP1	6.457453	7	7	7	1657	189.1	6.48	9409824	60
16418355	cyclic AMP-responsive element-binding protein 3-like protein 1	6.358382	2	2	2	519	57	5.17	7445190.8	43
4503937	protein NipSnap homolog 2 isoform 1	6.293706	1	1	1	286	33.7	9.36	7104313.8	0
194097365	pre-rRNA processing protein FTSJ3	6.257379	4	4	4	847	96.5	8.4	5769745.5	65
45359849	ras GTPase-activating protein-binding protein 2 isoform a	6.224066	2	2	2	482	54.1	5.55	3493208.4	32
111607441	lysine-rich nucleolar protein 1	6.113537	2	2	2	458	51.6	9.86	6270346.7	72
28173564	keratin, type II cytoskeletal 73	6.111111	3	8	1	540	58.9	7.23	42473201	131
28558979	mediator of RNA polymerase II transcription subunit 27 isoform 1	6.109325	1	1	1	311	35.4	9.31	3620249.3	25
4759208	serine/threonine-protein kinase TAO2 isoform 2	5.815062	5	5	3	1049	119.2	7.47	8399347.4	46
5031653	pre-mRNA-splicing factor SPF27	5.777778	1	1	1	225	26.1	5.66	33946300	0
29294639	mesoderm-specific transcript homolog protein isoform a	5.671642	1	1	1	335	38.8	9.74		24
7669526	pro-neuregulin-1, membrane-bound isoform isoform HRG-alpha	5.625	2	2	2	640	70.3	8.79	2625168.8	24
7705318	PTB domain-containing engulfment adapter protein 1 isoform a	5.592105	1	1	1	304	34.5	7.9	9819175.3	0
5803036	heterogeneous nuclear ribonucleoprotein A0	5.57377	1	1	1	305	30.8	9.29	1490759.8	28
31543164	THO complex subunit 6 homolog isoform 1	5.571848	1	1	1	341	37.5	7.43	2673409.7	0
4758138	probable ATP-dependent RNA helicase DDX5	5.537459	3	3	2	614	69.1	8.92	3597542	51
224028246	non-POU domain-containing octamer-binding protein isoform 1	5.52017	2	2	2	471	54.2	8.95	3004053.9	37
148746220	spermatogenesis-associated serine-rich protein 2	5.504587	2	2	2	545	59.5	8.9	3153847.6	36
46370095	AP-3 complex subunit mu-1	5.502392	1	1	1	418	46.9	6.93	6075176.8	0
7657269	sister chromatid cohesion protein PDS5 homolog B	5.459572	5	5	5	1447	164.6	8.47	4531284.6	49
54607086	selenocysteine-specific elongation factor	5.369128	2	3	2	596	65.3	8.35	13814800	39
215490011	aminoacyl tRNA synthase complex-interacting multifunctional protein 1 isoform b precursor	5.357143	2	3	2	336	37	8.65	4075510.7	39
53729346	plakophilin-1 isoform 1b	5.354752	4	4	4	747	82.8	9.13	5683643.6	76
319996735	oxysterol-binding protein-related protein 6 isoform c	5.31804	3	3	3	959	108.9	7.24	5385779.2	41
23110944	proteasome subunit alpha type-6	5.284553	1	1	1	246	27.4	6.76	7947643.4	33
331028739	chromatin target of PRMT1 protein isoform 2	5.220884	1	1	1	249	26.5	12.23	480099.84	0
4757834	BAG family molecular chaperone regulator 2	5.21327	1	1	1	211	23.8	6.7	4383120.7	0
28558977	mediator of RNA polymerase II transcription subunit 26	5.166667	2	2	2	600	65.4	9.16	6539048.2	21
284795266	signal recognition particle receptor subunit beta	5.166052	1	1	1	271	29.7	9.04	1662135.3	0
116812567	ras-related protein Rab-25	5.164319	1	1	1	213	23.5	5.96	19620138	0
296317324	guanine nucleotide-binding protein-like 3-like protein	5.154639	2	2	2	582	65.5	8.44	5922264.2	53
86991440	serine/arginine-rich splicing factor 5	5.147059	1	1	1	272	31.2	11.59	2401553.5	30
148806925	EGF, latrophilin and seven transmembrane domain-containing protein 1 precursor	5.072464	1	1	1	690	77.8	7.61		0
395455090	pre-mRNA-splicing regulator WTAP isoform 1	5.050505	1	1	1	396	44.2	5.19	1588340.1	0
6912486	U6 snRNA-associated Sm-like protein LSm4 isoform 1	5.035971	1	1	1	139	15.3	9.99	19962406	28

171906582	coiled-coil domain-containing protein 47 precursor	4.968944	1	1	1	483	55.8	4.87	7358645.8	0
7110715	SEC14-like protein 2 isoform 1	4.962779	1	1	1	403	46.1	7.84	159775589	0
6005882	serine protease 23 precursor	4.960836	1	1	1	383	43	9.42	5105948.7	20
18379334	RNA-binding protein with serine-rich domain 1	4.918033	1	1	1	305	34.2	11.84		16
156151381	THO complex subunit 7 homolog	4.901961	1	1	1	204	23.7	5.67	5190978.6	0
15529982	U3 small nucleolar ribonucleoprotein protein IMP4	4.810997	1	1	1	291	33.7	9.47	4194575.6	23
18375528	regulator of nonsense transcripts 3B isoform 1	4.761905	2	2	2	483	57.7	9.48	3117079	26
21361619	toll-interacting protein	4.744526	1	1	1	274	30.3	5.97		0
4506183	proteasome subunit alpha type-3 isoform 1	4.705882	1	1	1	255	28.4	5.33	3450667.5	54
6677723	replication factor C subunit 5 isoform 1	4.705882	1	1	1	340	38.5	7.2	1939970.5	22
26667177	39S ribosomal protein L46, mitochondrial	4.659498	1	1	1	279	31.7	7.05	3278240.2	31
190886435	ralBP1-associated Eps domain-containing protein 1 isoform a	4.654088	2	2	2	795	86.5	5.69	4490675.4	0
386781571	radixin isoform 1	4.635762	3	3	3	604	71	6.71	8260035.3	39
24431950	U4/U6 small nuclear ribonucleoprotein Prp4 isoform 1	4.597701	2	2	2	522	58.4	7.42	2413225.3	28
31317309	phosphatidylinositol 4-phosphate 5-kinase type-1 gamma isoform 2	4.491018	2	2	1	668	73.2	5.29	7449883.9	48
88501738	TRIO and F-actin-binding protein isoform 6	4.48203	8	8	8	2365	261.2	8.48	11264777	44
117647226	mitochondrial ribonuclease P protein 1 precursor	4.466501	1	1	1	403	47.3	9.36	7246249.5	0
7662284	protein-methionine sulfoxide oxidase MICAL2	4.448399	3	3	3	1124	126.6	8.65	3949788.1	31
347659028	isoform 1	4.434907	2	2	1	699	78.8	7.11	2714347.9	43
5453549	peroxiredoxin-4 precursor	4.428044	1	1	1	271	30.5	6.29	6074612.4	49
115430223	galectin-3 isoform 1	4.4	1	1	1	250	26.1	8.56	1134574.5	0
38683849	acidic fibroblast growth factor intracellular-binding protein isoform a	4.395604	1	1	1	364	41.9	6.48	7282587.8	0
16579828	E3 ubiquitin-protein ligase ZFP91 isoform 1	4.385965	1	1	1	570	63.4	7.36	1606522.4	0
21327708	nucleosome assembly protein 1-like 1	4.347826	1	1	1	391	45.3	4.46	4241253.1	0
4505753	phosphoglycerate mutase 1	4.330709	1	1	1	254	28.8	7.18	830452.96	0
133908629	SWI/SNF-related matrix-associated actin-dependent regulator of chromatin subfamily D member 1 isoform a	4.271845	1	1	1	515	58.2	9.25		15
223634006	ankyrin repeat domain-containing protein 65 isoform 1	4.260652	1	1	1	399	41.5	6.57	82139218	0
388240806	ribonuclease P protein subunit p38	4.240283	1	1	1	283	31.8	9.92	1159787.5	0
51702222	protein SPT2 homolog	4.233577	1	1	1	685	75.6	9.79		29
51477700	mediator of RNA polymerase II transcription subunit 15 isoform a	4.187817	2	3	2	788	86.7	9.42	3243658.1	0
4506193	proteasome subunit beta type-1	4.149378	1	1	1	241	26.5	8.13		32
301897477	beta-enolase isoform 1	4.147465	1	1	1	434	47	7.71	18775423	57
150417989	cyclin-K	4.137931	1	1	1	580	64.2	8.41	2625434	0
157426877	RNA 3'-terminal phosphate cyclase-like protein	4.021448	1	1	1	373	40.8	9.26	4406411.9	42
495528154	collagen alpha-1(V) chain isoform 2 preproprotein	3.971708	4	4	4	1838	183.5	5.07	9012422.6	17
14110420	heterogeneous nuclear ribonucleoprotein D0 isoform a	3.943662	1	1	1	355	38.4	7.81	6847063.3	0
429535832	telomeric repeat-binding factor 2	3.874539	1	1	1	542	59.6	9.35	1275454.7	20
374717343	tricarboxylate transport protein, mitochondrial isoform b	3.773585	1	1	1	318	35	9.99	15746364	34
169167792	PREDICTED: uncharacterized protein LOC401180	3.75	1	1	1	320	33.3	11.02	10955426	0

304555583	elongation factor 1-delta isoform 1	3.709428	1	1	1	647	71.4	6.42	2697130.2	27
50513245	chromatin assembly factor 1 subunit A	3.661088	2	2	2	956	106.8	5.94	7632923.3	37
330864679	F-actin-capping protein subunit beta isoform 2	3.610108	1	1	1	277	31.3	5.59	8651740.1	0
237820620	glutamate-rich WD repeat-containing protein 1	3.587444	1	1	1	446	49.4	4.92	6672196.4	25
25092725	ribosomal RNA-processing protein 7 homolog A	3.571429	1	1	1	280	32.3	9.58	5126816.8	30
190610012	zinc finger protein 24	3.532609	1	1	1	368	42.1	6.21	870445.24	0
73760405	thymopoietin isoform beta	3.524229	1	1	1	454	50.6	9.38	5108804.4	42
50959115	THO complex subunit 5 homolog	3.513909	1	1	1	683	78.5	6.87	4316529.2	0
5453597	F-actin-capping protein subunit alpha-1	3.496503	1	1	1	286	32.9	5.69	10498502	37
28460688	alpha-taxilin	3.479853	1	1	1	546	61.9	6.52		0
54112117	splicing factor 3B subunit 1 isoform 1	3.374233	3	3	3	1304	145.7	7.09	3253405.9	51
5453998	importin-7	3.371869	3	3	3	1038	119.4	4.82	4379258.4	61
14150141	programmed cell death protein 2-like	3.351955	1	1	1	358	39.4	4.86	14198839	28
112807226	ELM2 and SANT domain-containing protein 1	3.349282	1	2	1	1045	114.9	9.19	9555134.1	18
6031192	phosphate carrier protein, mitochondrial isoform a precursor	3.314917	1	1	1	362	40.1	9.38	12020263	40
325296984	replication factor C subunit 1 isoform 2	3.310105	3	3	3	1148	128.2	9.36	5781853.3	38
4504511	dnaJ homolog subfamily A member 1	3.274559	1	2	1	397	44.8	7.08		0
68509926	DHX15	3.27044	2	2	2	795	90.9	7.46	2611454.3	30
5803187	transaldolase	3.264095	1	1	1	337	37.5	6.81	3783603.7	28
21071067	transcription initiation factor TFIID subunit 5	3.25	2	2	2	800	86.8	5.64	2301519.5	40
222136634	tetratricopeptide repeat protein 26 isoform 1	3.249097	1	1	1	554	64.1	6.93	36886425	0
188528686	protein-lysine methyltransferase METTL21A	3.211009	1	1	1	218	24.6	6.27	3789709.6	32
4505571	sequestosome-1 isoform 1	3.181818	1	1	1	440	47.7	5.22	2515987.9	0
21264355	SWI/SNF-related matrix-associated actin-dependent regulator of chromatin subfamily E member 1	3.163017	1	1	1	411	46.6	4.88	8316808.2	0
18375676	regulator of nonsense transcripts 2	3.144654	4	4	4	1272	147.7	5.69	5518080.4	33
88853069	vitronectin precursor	3.138075	1	1	1	478	54.3	5.8	12249345	41
10280622	alpha-amylase 2B precursor	3.131115	1	1	1	511	57.7	7.09	1300768.3	44
215490049	mortality factor 4-like protein 2	3.125	1	1	1	288	32.3	9.72	6268368.3	27
384229051	thymidine phosphorylase isoform 2 proprotein	3.080082	1	1	1	487	50.4	5.53	1445298.7	31
154426310	SPATS2-like protein isoform a	3.046595	1	1	1	558	61.7	9.64	3405073	0
4503481	elongation factor 1-gamma	2.974828	1	1	1	437	50.1	6.67	874122.28	30
7662394	rab11 family-interacting protein 2	2.929688	1	1	1	512	58.2	9.32	4113930.1	0
38348348	carabin isoform 1	2.914798	1	1	1	446	49.7	8.76	5833110.5	38
56699482	protein FAM98A	2.895753	1	1	1	518	55.2	8.95	1000443.6	43
92110027	glutaminyl-peptide cyclotransferase-like protein isoform 1	2.879581	1	1	1	382	42.9	9.82	3682631.1	29
20806097	nucleolar complex protein 3 homolog	2.875	1	1	1	800	92.5	9.17		0
32171205	calcium uptake protein 3, mitochondrial	2.830189	1	1	1	530	60.7	8.21		28
5174513	mothers against decapentaplegic homolog 3 isoform 1	2.823529	1	1	1	425	48.1	7.15	7026955.7	0
24234688	stress-70 protein, mitochondrial precursor	2.798233	1	1	1	679	73.6	6.16		21

7019547	serine/threonine-protein kinase TBK1	2.743484	1	1	1	729	83.6	6.79	69338556	0
32484979	AP-3 complex subunit beta-1 isoform 1	2.74223	2	2	2	1094	121.2	6.04	6312359.9	54
54607091	sentrin-specific protease 2	2.716469	1	1	1	589	67.8	9.48	1663196.4	0
12408654	galactosylgalactosylxylosylprotein 3-beta-glucuronosyltransferase 3	2.686567	1	1	1	335	37.1	8.27	16707314	0
387849350	membrane-spanning 4-domains subfamily A member 14 isoform 4	2.668539	1	1	1	712	80.1	5.55	36064359	0
325651836	SWI/SNF-related matrix-associated actin-dependent regulator of chromatin subfamily A member 5	2.661597	3	3	1	1052	121.8	8.09	7409464.3	53
21237805	SWI/SNF complex subunit SMARCC2 isoform a	2.635914	3	3	1	1214	132.8	5.69	2988628.9	49
31377806	polymeric immunoglobulin receptor precursor	2.617801	1	1	1	764	83.2	5.74	3526801.7	26
116063568	torsin-4A	2.600473	1	1	1	423	46.9	9.94	1727292.8	0
29029601	probable ATP-dependent RNA helicase DHX37	2.592913	2	2	2	1157	129.5	8.1	2023163.4	38
39932583	protein NRDE2 homolog	2.57732	2	2	2	1164	132.6	7.68	2924654.6	34
313760643	SUN domain-containing protein 2 isoform a	2.574526	1	1	1	738	82.5	6.47	1161379.3	24
259013556	fragile X mental retardation syndrome-related protein 2	2.526003	1	1	1	673	74.2	6.23	371794.91	0
62988322	testis-specific Y-encoded-like protein 1	2.517162	1	1	1	437	49.2	5.45	5154199.7	32
48762926	periodic tryptophan protein 2 homolog	2.50272	2	2	2	919	102.4	6.15	2049686.4	42
7661952	squamous cell carcinoma antigen recognized by T-cells 3	2.492212	1	1	1	963	109.9	5.57	1977723.7	0
166706887	syncoilin isoform 1	2.489627	1	1	1	482	55.2	4.61	77157547	0
293336691	histone deacetylase 2	2.459016	1	1	1	488	55.3	5.91	4614488.4	0
192807323	transcription activator BRG1 isoform A	2.44193	3	3	3	1679	188	8.19	2603356.3	0
51599156	chromodomain-helicase-DNA-binding protein 4	2.405858	3	4	3	1912	217.9	5.86	8728393.5	49
4759276	U3 small nucleolar RNA-interacting protein 2	2.315789	1	1	1	475	51.8	7.85	4107213.6	0
19923640	ribosome-releasing factor 2, mitochondrial isoform 1	2.310655	1	1	1	779	86.5	6.51	27143905	35
19923907	oxidoreductase NAD-binding domain-containing protein 1 precursor	2.24359	1	1	1	312	34.8	8.37	22456142	34
55770832	cerebellar degeneration-related protein 2	2.202643	1	1	1	454	51.8	5.06	2620985.5	46
30578410	STT3B	2.179177	2	2	2	826	93.6	8.91	4986021.1	31
7657015	tRNA-splicing ligase RtcB homolog	2.178218	1	1	1	505	55.2	7.23	2706404.5	21
5730037	exocyst complex component 5	2.118644	1	1	1	708	81.8	6.71	5328331.9	0
14249338	BUD13 homolog isoform 1	2.100162	1	1	1	619	70.5	9.86	2374269	28
23397574	sorting nexin-33	2.090592	1	1	1	574	65.2	6.79	2451281.6	46
110227613	arf-GAP with GTPase, ANK repeat and PH domain-containing protein 3 isoform a	2.08562	1	1	1	911	97.9	7.96	3135478.1	40
157817023	cyclin-dependent kinase 12 isoform 1	2.080537	2	2	2	1490	164.1	9.44	1828329.3	0
5032179	transcription intermediary factor 1-beta	2.035928	1	1	1	835	88.5	5.77	503749.73	28
217035121	HMG box transcription factor BBX isoform 1	2.019129	2	2	2	941	105.1	8.79	3239596.7	41
167234419	thyroid hormone receptor-associated protein 3	1.989529	1	1	1	955	108.6	10.15	1606042.6	0
126723149	cactin	1.978892	1	1	1	758	88.6	9.14	293438.36	0
41055989	M-phase phosphoprotein 8	1.976744	1	1	1	860	97.1	6.06	4905866	29
303305009	peptidyl-prolyl cis-trans isomerase E isoform 4	1.910828	1	1	1	314	35	6.38	4640414.3	36
296531349	histone lysine demethylase PHF8 isoform 1	1.886792	1	1	1	1060	117.8	8.72	70491.92	29

9966799	something about silencing protein 10	1.878914	1	1	1	479	54.5	5.62	8918665.8	50
4504699	inhibin beta A chain precursor	1.877934	1	1	1	426	47.4	8.03	8913677.4	41
4557565	DNA excision repair protein ERCC-6	1.875419	2	2	2	1493	168.3	8.09	6140707.2	46
27436957	membrane-associated guanylate kinase, WW and PDZ domain-containing protein 2	1.85567	1	1	1	1455	158.7	6.35	39581229	0
25777600	26S proteasome non-ATPase regulatory subunit 1 isoform 1	1.783841	1	2	1	953	105.8	5.39	16934454	0
508772597	annexin A11 isoform 1	1.782178	1	1	1	505	54.4	7.65	17827428	0
104487006	receptor-type tyrosine-protein phosphatase S isoform 1 precursor	1.74538	1	1	1	1948	216.9	6.46		0
13325075	sulfhydryl oxidase 1 isoform a precursor	1.740295	1	1	1	747	82.5	8.92	2005398.7	0
164519146	transcriptional repressor p66-alpha	1.737757	1	1	1	633	68	9.94	3070744.1	43
325053640	cell cycle progression protein 1 isoform 2	1.73482	1	1	1	807	93.4	6.34	1931763.3	0
212276096	leucine-rich repeat flightless-interacting protein 1 isoform 3	1.732673	1	1	1	808	89.2	4.65	729127.23	0
5032087	splicing factor 3A subunit 1 isoform 1	1.639344	1	1	1	793	88.8	5.22	4832724.6	0
87196339	collagen alpha-1(VI) chain precursor	1.55642	1	1	1	1028	108.5	5.43	2342169.6	18
42558250	caprin-1 isoform 1	1.551481	1	1	1	709	78.3	5.25	2151951.6	37
38505218	acyl-CoA dehydrogenase family member 11	1.538462	1	1	1	780	87.2	8.02	11865715	34
32307144	procollagen-lysine,2-oxoglutarate 5-dioxygenase 1 precursor	1.513067	1	1	1	727	83.5	6.95	2330727.3	0
5454064	RNA-binding protein 14 isoform 1	1.494768	1	1	1	669	69.4	9.67	1044258.4	0
13514831	probable ATP-dependent RNA helicase DDX10	1.485714	1	1	1	875	100.8	8.63	2232121.9	28
21281677	WD repeat-containing protein 36	1.472135	1	1	1	951	105.3	7.53	2655290.8	33
4505939	DNA-directed RNA polymerase II subunit RPB1	1.42132	1	1	1	1970	217.1	7.37	2615288.2	45
124256489	crooked neck-like protein 1	1.415094	1	1	1	848	100.4	8	247709.48	0
444909146	ESF1 homolog	1.410106	1	1	1	851	98.7	5.11	20216219	0
54792088	GLTSCR1-like protein	1.390176	1	1	1	1079	115	6.87		0
4505941	DNA-directed RNA polymerase II subunit RPB2	1.362862	1	1	1	1174	133.8	6.87	1402823	0
14043022	methionine--tRNA ligase, cytoplasmic	1.333333	1	1	1	900	101.1	6.16	5938334.7	40
45238858	WD repeat-containing protein 44 isoform 1	1.314348	1	1	1	913	101.3	5.45		0
116805340	glycine--tRNA ligase precursor	1.217862	1	1	1	739	83.1	7.03	7049465.1	23
48255900	probable global transcription activator SNF2L2 isoform a	1.194969	1	1	1	1590	181.2	7.2	2213361.7	0
28558971	mediator of RNA polymerase II transcription subunit 23 isoform a	1.169591	1	1	1	1368	156.4	7.4	1896919.5	36
311893365	calpain-1 catalytic subunit	1.120448	1	1	1	714	81.8	5.67	4489343	29
284172420	prolyl endopeptidase-like isoform 1	1.100413	1	1	1	727	83.9	6.38		0
4502951	collagen alpha-1(III) chain preproprotein	1.091405	1	1	1	1466	138.5	6.57	1754478.6	27
239735519	myotubularin-related protein 5	1.056524	1	1	1	1893	210.9	7.11	2936989.3	0
28872761	myotubularin-related protein 1	1.052632	1	1	1	665	74.6	7.14	2073944.5	36
194097392	Golgin subfamily A member 2	0.998004	1	1	1	1002	113	5.02	5905829.4	38
38788274	nucleosome-remodeling factor subunit BPTF isoform 1	0.924658	1	1	1	2920	324.9	7.08	2643598	82
32967603	bromodomain adjacent to zinc finger domain protein 1A isoform a	0.899743	1	1	1	1556	178.6	6.6	1135478	35
10863903	E3 ubiquitin-protein ligase TRIP12	0.853414	1	1	1	1992	220.3	8.48	899335.98	37
472235318	formin-1 isoform a	0.845666	1	1	1	1419	157.5	8.44	6822721.2	0

410169893	PREDICTED: fibrosin-like 1, partial	0.77951	1	1	1	898	95.7	9.22		0
154354979	unconventional myosin-X	0.728863	1	1	1	2058	237.2	6.21		0
194018488	peroxisome biogenesis factor 6	0.714286	1	1	1	980	104	6.34	4001311.5	42
14719829	NACHT, LRR and PYD domains-containing protein 1 isoform 1	0.678887	1	1	1	1473	165.8	6.84	1902120.8	0
357933602	zinc finger protein 638 isoform 1	0.65723	1	1	1	1978	220.5	6.38	275337	0
380692334	nuclear envelope pore membrane protein POM 121 isoform 1	0.600601	1	1	1	999	100.8	9.83	4307883.3	36
31563330	A-kinase anchor protein 13 isoform 1	0.56798	1	1	1	2817	307.7	5.2	1064150.1	33
89276766	trophinin isoform 5	0.55905	1	1	1	1431	143.6	9.03	3068874.1	32
114842389	myosin-7B	0.504286	1	1	1	1983	225.7	6.14		30
28559039	mediator of RNA polymerase II transcription subunit 1	0.442758	1	1	1	1581	168.4	8.73	1723173.6	27
33620769	E3 ubiquitin-protein ligase RBBP6 isoform 1	0.390625	1	1	1	1792	201.4	9.64	4045513	39
126012571	basement membrane-specific heparan sulfate proteoglycan core protein precursor	0.318834	1	1	1	4391	468.5	6.51	18449121	0
403310693	nebulin isoform 4	0.186916	1	1	1	8560	990.2	9.01	3630608.1	0
